# The blood flow-*klf6a-tagln2* axis drives vessel pruning in zebrafish by regulating endothelial cell rearrangement and actin cytoskeleton dynamics

**DOI:** 10.1371/journal.pgen.1009690

**Published:** 2021-07-28

**Authors:** Lin Wen, Tao Zhang, Jinxuan Wang, Xuepu Jin, Muhammad Abdul Rouf, Desha Luo, Yuan Zhu, Daoxi Lei, Hans Gregersen, Yeqi Wang, Guixue Wang

**Affiliations:** 1 Key Laboratory for Biorheological Science and Technology of Ministry of Education, Bioengineering College of Chongqing University, Chongqing, China; 2 State and Local Joint Engineering Laboratory for Vascular Implants, Bioengineering College of Chongqing University, Chongqing, China; Fred Hutchinson Cancer Research Center, UNITED STATES

## Abstract

Recent studies have focused on capillary pruning in various organs and species. However, the way in which large-diameter vessels are pruned remains unclear. Here we show that pruning of the zebrafish caudal vein (CV) from ventral capillaries of the CV plexus in different transgenic embryos is driven by endothelial cell (EC) rearrangement, which involves EC nucleus migration, junction remodeling, and actin cytoskeleton remodeling. Further observation reveals a growing difference in blood flow velocity between the two vessels in CV pruning in zebrafish embryos. With this model, we identify the critical role of Kruppel-like factor 6a (*klf6a*) in CV pruning. Disruption of *klf6a* functioning impairs CV pruning in zebrafish. *klf6a* is required for EC nucleus migration, junction remodeling, and actin cytoskeleton dynamics in zebrafish embryos. Moreover, actin-related protein transgelin 2 (*tagln2*) is a direct downstream target of *klf6a* in CV pruning in zebrafish embryos. Together these results demonstrate that the *klf6a*-*tagln2* axis regulates CV pruning by promoting EC rearrangement.

## Author summary

Vascular remodeling is critical for vascular physiology and pathology. The primitive vascular plexus formed by angiogenesis, subsequently undergoes extensive vascular remodeling to establish a functionally and hierarchically branched network of blood vessels. Vascular remodeling mainly consists of vessel pruning and fusion. Endothelial cell rearrangement plays an essential role in vessel pruning, which involves endothelial cell migration and polarity. Dysfunction of flow-induced vascular remodeling will cause arteriovenous malformation and impair reperfusion of ischemia stroke. In this study, we show that the large-diameter vessel of the caudal vein is pruned from ventral capillaries of the caudal vein plexus in zebrafish embryos. With this model, we observe a growing difference in blood flow velocity between two branches in vessel pruning. We identify that the *klf6a*-*tagln2* axis regulates CV pruning by promoting endothelial cell rearrangement and junction remodeling. Our results suggest that the caudal vein formation is an ideal model for screening the potential genes involved in vascular remodeling-related disease.

## Introduction

Primitive vascular beds are often formed by angiogenesis, which is defined as the sprouting of new blood vessels from existing ones [1]. To adapt to supplies of oxygen and nutrients, the primitive vascular plexus remodels itself and eventually develops into a functionally and hierarchically branched network of blood vessels. During vascular remodeling, a subset of local vessels is pruned away by active regression [2], whereas other microvessels fuse into larger diameter vessels in response to hemodynamic force [3].

Vascular pruning has been described for many vascular beds, such as the vasculature of the mouse retina and venous intersegmental vessels (vISVs) [2], midbrain vasculature [4], eye cranial division of the internal carotid artery (CrDI) [5] and subintestinal veins (SIVs) [6] of the zebrafish. It appears that pruning occurs preferentially in vascular loops with different branches. Flow-induced endothelial cell (EC) rearrangement plays an essential role in vascular pruning [2]. Flow-induced EC rearrangement involves EC migration, flow-induced EC polarity, junction remodeling [7–9]. Researchers have used live images to monitor vascular remodeling and simultaneously measure the velocity of blood flow [4,5,10,11]. Changes in blood flow inhibit EC rearrangement and vascular pruning in different vascular beds. Several signaling systems have been reported to regulate these processes. For example, integrin signaling is required for shear stress-induced EC migration [12]. Endoglin inhibits vascular malformation by regulating flow-induced cell migration through vascular endothelial growth factor receptor 2 (VEGFR2) signaling [13]. GTPase ras homolog family member A (RhoA) and ras-related C3 botulinum toxin substrate 1 (Rac1) are essential for shear stress-induced EC polarization [14]. Apelin signaling is involved in flow-induced endothelial polarity in zebrafish and *in vitro* [15]. Partition-defective 3 (PAR-3) responds to laminar flow to control endothelial polarity and vascular inflammation [16], and dynamic VE-cadherin in cell-cell junctions is involved in vessel pruning [2,6]. Phosphatidyl inositol 3-kinase (PI3K) signaling prevents actomyosin contractility to regulate EC rearrangement during vascular development [17]. However, the precise role of blood flow in vessel pruning remains unclear.

In this study we used time-lapse live imaging of several transgenic reporter lines to show that the zebrafish caudal vein (CV) is remodeled from ventral capillaries of the CV plexus (CVP) by vessel pruning. Using this model, we investigated how hemodynamic forces regulate vessel pruning. Our results suggest that the magnitude of the difference in blood flow between two branches can contribute to vascular stabilization/pruning. Moreover, the results of live imaging show that junction remodeling and actin cytoskeleton dynamics are involved in CV pruning. We show that the Kruppel-like factor 6a (*klf6a*)-actin-related protein transgelin 2 (*tagln2*) axis drives CV pruning in zebrafish embryos by regulating EC rearrangement.

## Results

### CV formation is accompanied by a decrease in vascular loops in zebrafish embryos

**Fig 1:**
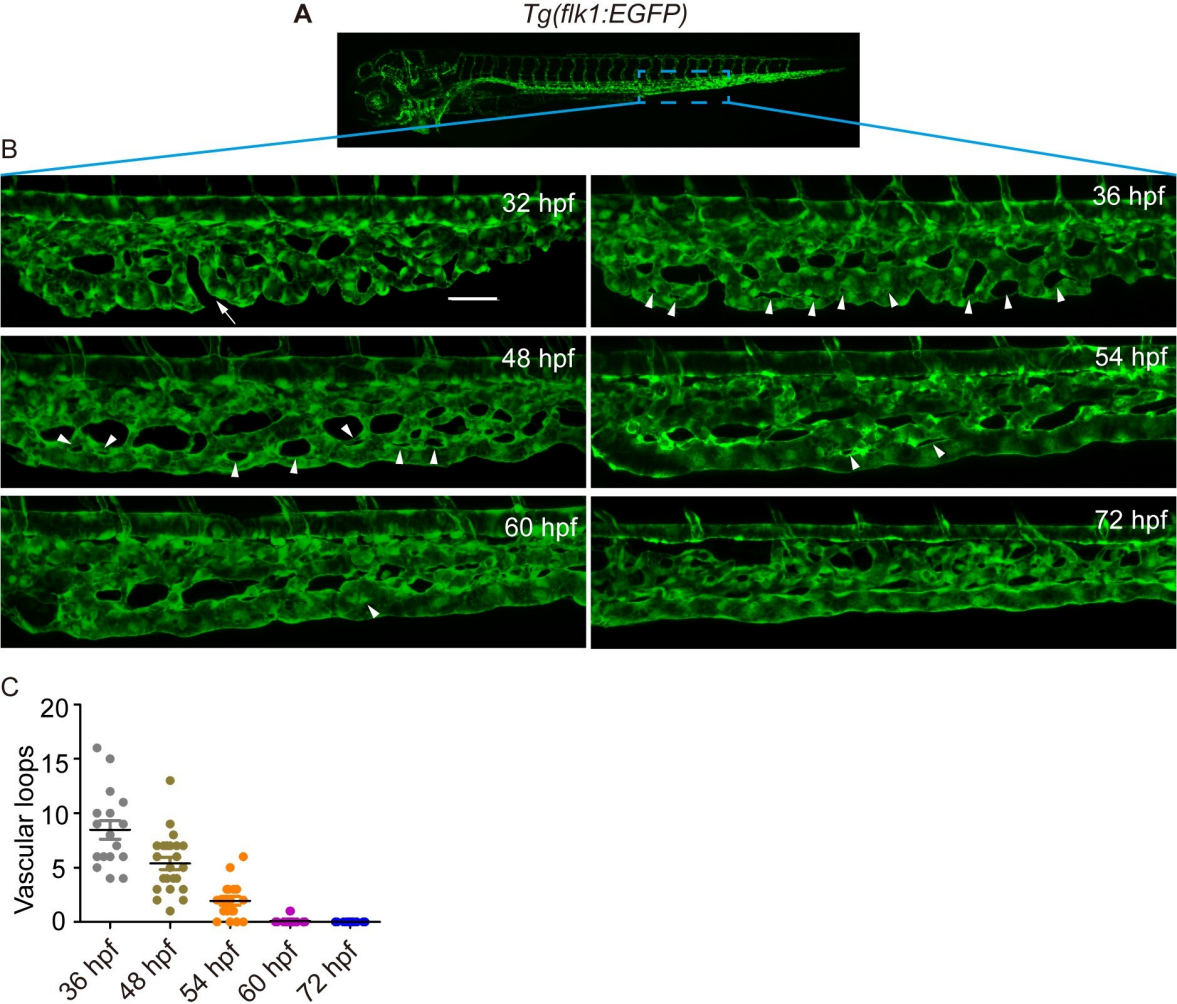
The zebrafish caudal vein formation involves vascular remodeling. (A) A sketch map of the region. (B) Confocal images of the caudal vasculature of zebrafish embryos. The arrow points to ventral CVP capillaries without connection. Arrowheads point to vascular loops. Scale bar: 50 μm. (C) Quantification of the number of vascular loops in the CV: 36 hpf, n = 17 embryos; 48 hpf, n = 23 embryos; 54 hpf, n = 19 embryos; 60 hpf, n = 22 embryos; 72 hpf, n = 13 embryos.

Ventral capillaries of the CVP develop into the CV during the development of zebrafish embryos [18–20]. However, the details of this developmental process and its underlying mechanism remain unclear. To address this, we used confocal microscopy to observe CV formation in wild-type (WT) *Tg(flk1*: *EGFP)* transgenic live embryos from 32 h post fertilization (hpf) to 72 hpf (Fig 1A). We found that CV formation is a dynamic process and that the CV is remodeled from the ventral capillaries of the CVP in zebrafish embryos. As shown in Fig 1B, the ventral capillaries of the CVP migrated horizontally and connected with each other at 32 hpf. At 36 hpf, they developed into luminal vessels with vascular loops. As a result of blood flow, these luminal vessels went through a dramatic remodeling process and developed into the CV at 60 hpf. It is interesting that there was a negative correlation between the number of vascular loops (white arrowheads) and CV formation. As shown in Fig 1C, as the number of vascular loops decreased from 8.47 at 36 hpf to 2 at 54 hpf, the ventral capillaries of the CVP eventually rearranged to establish the uniform vessel called the CV at 60 hpf. Together the remodeling of the ventral capillaries of the CVP to establish the CV is accompanied by a decrease in vascular loops in zebrafish embryos.

### Vessel pruning drives CV formation in zebrafish embryos

**Fig 2:**
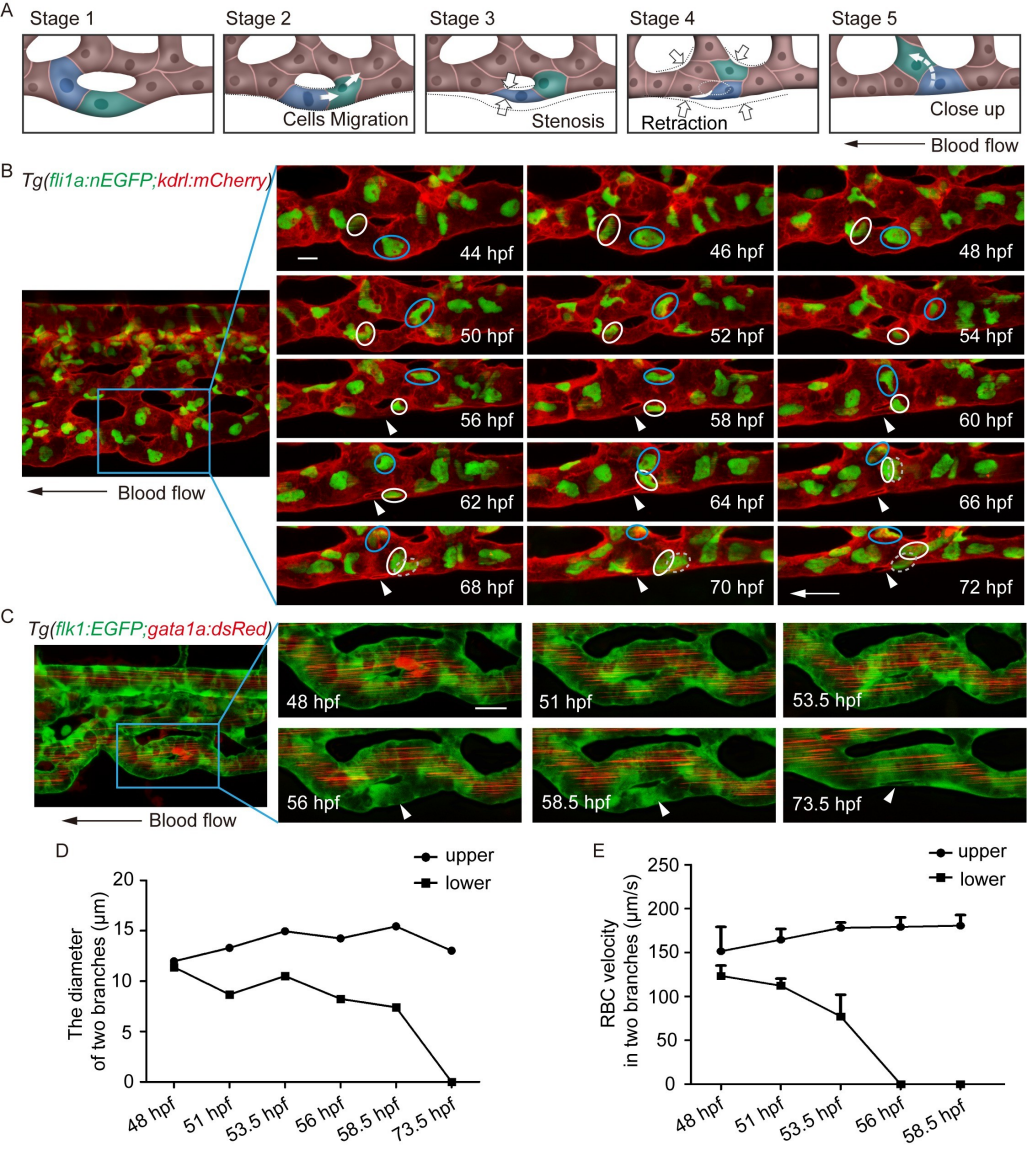
Endothelial cell behavior and the growing difference in blood flow between two branches in vessel pruning. (A) Sketch map of EC rearrangement during vessel pruning. (B) Time-lapse live imaging of *Tg(fli1a*:*nEGFP;kdrl*:*mCherry)* embryos shows EC nucleus migration in CV pruning. Arrowheads indicate the pruned vessel. The arrows indicate the direction of the blood flow. Colored circles indicate ECs nuclei. 24 time-lapse live imaging were taken. Scale bar: 10 μm. (C) Time-lapse live imaging of *Tg(flk1*:*EGFP;gata1a*:*dsRed)* embryos shows vessel stenosis and the change of blood flow in CV pruning. Arrowheads indicate the lack of blood flow in the regressing vessel. The arrow indicates the direction of the blood flow. 10 time-lapse live imaging were taken. Scale bar: 10 μm. (D) The diameters of two branches at the indicated stages. (E) The velocity of RBCs in two branches at the indicated stages (48 hpf: S1 Video, 51 hpf: S2 Video, 53.5 hpf: S3 Video, 56 hpf: S4 Video, 58.5 hpf: S5 Video). The number of RBCs is calculated for blood flow at each stage: upper branch, 48.5 hpf: n = 5, 51 hpf: n = 10, 53.5 hpf: n = 8, 56 hpf: n = 9, 58.5 hpf: n = 10; lower branch, 48.5 hpf: n = 10, 51 hpf: n = 9, 53.5 hpf: n = 2.

The gradual decrease in the number of vascular loops during CV formation in zebrafish resembles the vessel pruning described in the mouse retina [2]. During the pruning of vascular loops in the mouse retina model, one branch is stable, whereas the other is pruned by flow-induced EC rearrangement. Here we hypothesized that the gradual decrease in vascular loops during CV formation in zebrafish is regulated by vessel pruning. To validate this hypothesis, we used time-lapse live imaging to monitor the remodeling of vascular loops during CV formation in zebrafish embryos of the *Tg(fli1a*:*nEGFP;kdrl*:*mCherry)* line (Fig 2B). By analyzing these time-lapse imaging sequences (14 videos), we found that stabilization/regression of competitive branches occurred during CV formation in zebrafish and that EC nucleus migration and vessel stenosis were involved in the regression of vessel branches (Fig 2A and 2B). As shown in Fig 2B, the diameters of the two branches were initially comparable at 44 hpf (stage 1 in Fig 2A). Subsequently, the lower branch became stenotic and eventually regressed, whereas the upper one remained stable.

In the lower branch, the EC nucleus (shown in blue) migrated against the direction of the blood flow from 44 to 50 hpf (stage 2 in Fig 2A), followed by a rapid stenosis of the branch at 50 hpf (stage 3 in Fig 2A). Later on, this EC nucleus (shown in blue) continued to move forward from 50 to 54 hpf (stage 4 in Fig 2A), and then it moved around to the upper branch from 54 to 72 hpf (stage 5 in Fig 2A). Meanwhile, another EC nucleus (shown in white) also moved against the blood flow from 52 to 56 hpf (stage 2 in Fig 2A). The lower branch became stenotic once again at 58 hpf (stage 3 in Fig 2A). Subsequently, the EC nucleus (shown in white) divided into two daughters at 66 hpf, and one daughter continued to move forward from 68 to 72 hpf (stage 4 in Fig 2A). At 72 hpf, the lower branch was almost regressed (stage 5 in Fig 2A).

To further observe EC migration in the regressing branch during CV formation in zebrafish embryos, we generated the knock-in (KI) fish *KI(Cdh5-mRFP)* using CRISPR/Cas9 technology. An *mRFP* sequence was inserted into the last exon (exon 12) of the *cdh5* gene before the stop codon and fused into *cdh5-mRFP*, which was identified by PCR of its genomic DNA using target site-specific and donor-specific primers and subsequent sequencing analysis (S1A–S1C Fig). To observe the behavior of ECs in branch stenosis, we performed live imaging of *Tg(fli1a*:*nEGFP);KI(Cdh5-mRFP)* embryos. We found that both of EC nucleus migration and dynamic changes in cytoplasm contributed to vessel stenosis during CV formation in zebrafish embryos (S1D Fig). The EC nucleus (shown in white) migrated with the blood flow from 48 to 50 hpf. In contrast, the EC nucleus (shown in blue) migrated against the blood flow from 50 to 60 hpf. As the EC nuclei migration, the retraction of cytoplasmic extension can be observed.

EC polarity against the blood flow plays a pivotal role in vessel pruning [2]. To determine whether EC polarity against the blood flow is involved in the remodeling of the ventral capillaries of the CVP into the CV, we generated the transgenic zebrafish embryo line *Tg(fli1a*:*EGFP;fli1a*:*B4GALT1-mCherry)* following a previous report [15]. As shown in S2A Fig, the ECs of arterial intersegmental vessels (aISVs) became polarized at 48 hpf, in which the position of Golgi is relative to the nucleus against the blood flow. We then performed time-lapse live imaging to trace EC polarities within the regressing branch during CV formation in WT zebrafish embryos from 46 to 52 hpf (S2B Fig). We found that, among nine moves, 19 out of 22 venous ECs did not reveal polarities against the blood flow during zebrafish CV formation, which is consistent with the previous study [15].

Taken together, our results demonstrate that CV formation is a process of vessel pruning (hereafter, “CV pruning”).

### There is a growing difference in blood flow velocity between neighboring vessels in CV pruning

Previous researchers presumed that the growing difference in blood flow velocity between neighboring branches determines vessel pruning [2]. To validate this hypothesis, we performed time-lapse live imaging of *Tg(flk1*:*EGFP;gata1*:*dsRed)* embryos to monitor dynamic changes in both blood flow velocity and diameter within neighboring branches of vascular loops in CV pruning (Fig 2C). We found that the difference in blood flow velocity increased until one branch was pruned. At 48 hpf, the diameter of the upper and lower branches did not differ, and the blood flow velocity was comparable between the two branches (Fig 2C–2E). Subsequently, the lower branch became stenotic as the diameter decreased and regressed at 73.5 hpf (Fig 2C and 2D). This regression was accompanied by a decrease in blood flow velocity until blood flow ceased at 56 hpf (Fig 2C and 2E). In contrast, the upper branch became stable and showed a gradual increase in blood flow velocity in this period. These results reveal a growing difference in blood flow velocity between neighboring branches during CV pruning that may trigger vessel pruning.

To further validate the effect of blood flow on CV pruning in zebrafish embryos, we treated *Tg(flk1*:*EGFP;gata1a*:*dsRed)* embryos with a low concentration of tricaine (0.06 mg/mL) from 30 to 72 hpf. This dose of tricaine did not affect the morphology of the embryos (S3A Fig) but only decreased the velocity of the red blood cells (RBCs; S3E Fig). Compared to untreated WT embryos, embryos treated with tricaine showed more vascular loops in the CV at 72 hpf (average loops: control = 0.26087, tricaine = 1.32143; S3B and S3F Fig). We also validated the effect of blood flow on CV pruning by injecting a low dose of *tnnt2a* morpholino (MO; 0.2 ng) into zebrafish embryos at the single-cell stage. This dose of *tnnt2a* MO did not affect the morphology (S3C Fig) of the embryos but decreased the velocity of the RBCs (S3G Fig) in the CV. Injection with the low concentration of *tnnt2a* MO also increased the number of vascular loops in the CV at 72 hpf (average loops: control = 0.41666, tricaine = 1.102; S3D and S3H Fig).

Taken together, these results show that reduced blood flow inhibits CV pruning in zebrafish embryos.

### *Klf6a* is responsive to blood flow and is expressed in the CV of zebrafish embryos

The Kruppel-like factor (Klf) family of transcription factors plays important roles in regulating hemodynamic force-mediated cardiovascular homeostasis [21]. Of 24 Klf family genes in zebrafish, only *klf6a* was enriched in the CVP of zebrafish embryos at 36 hpf by whole-mount in situ hybridization (WISH) [22]. Therefore, we speculated that *klf6a* could be involved in CV pruning in zebrafish embryos. Consistent with previous reports [22,23], our WISH results showed that *klf6a* was indeed enriched in the zebrafish CVP region at 36 hpf compared to *klf2a*, *klf2b*, *klf4*, and *klf6b* (S4A Fig). To determine whether *klf6a* responds to blood flow, we performed a flow chamber experiment in which we exposed human umbilical vein ECs (HUVECs) to static (0 dyn/cm^2^) or physiological shear stress (12 dyn/cm^2^) for 12 h. Our results revealed that flow shear stress (FSS) induces KLF6 expression at both the mRNA (S4B Fig) and protein (S4C Fig) levels in HUVECs *in vitro*. Furthermore, WISH revealed that reductions in the velocity of blood flow due to treatment with tricaine and *tnnt2a* MO significantly disrupted *klf6a* mRNA level in the CVP region at 36 hpf (S4D and S4E Fig). These results suggest that *klf6a* is responsive to hemodynamic force both *in vitro* and *in vivo*.

**Fig 3:**
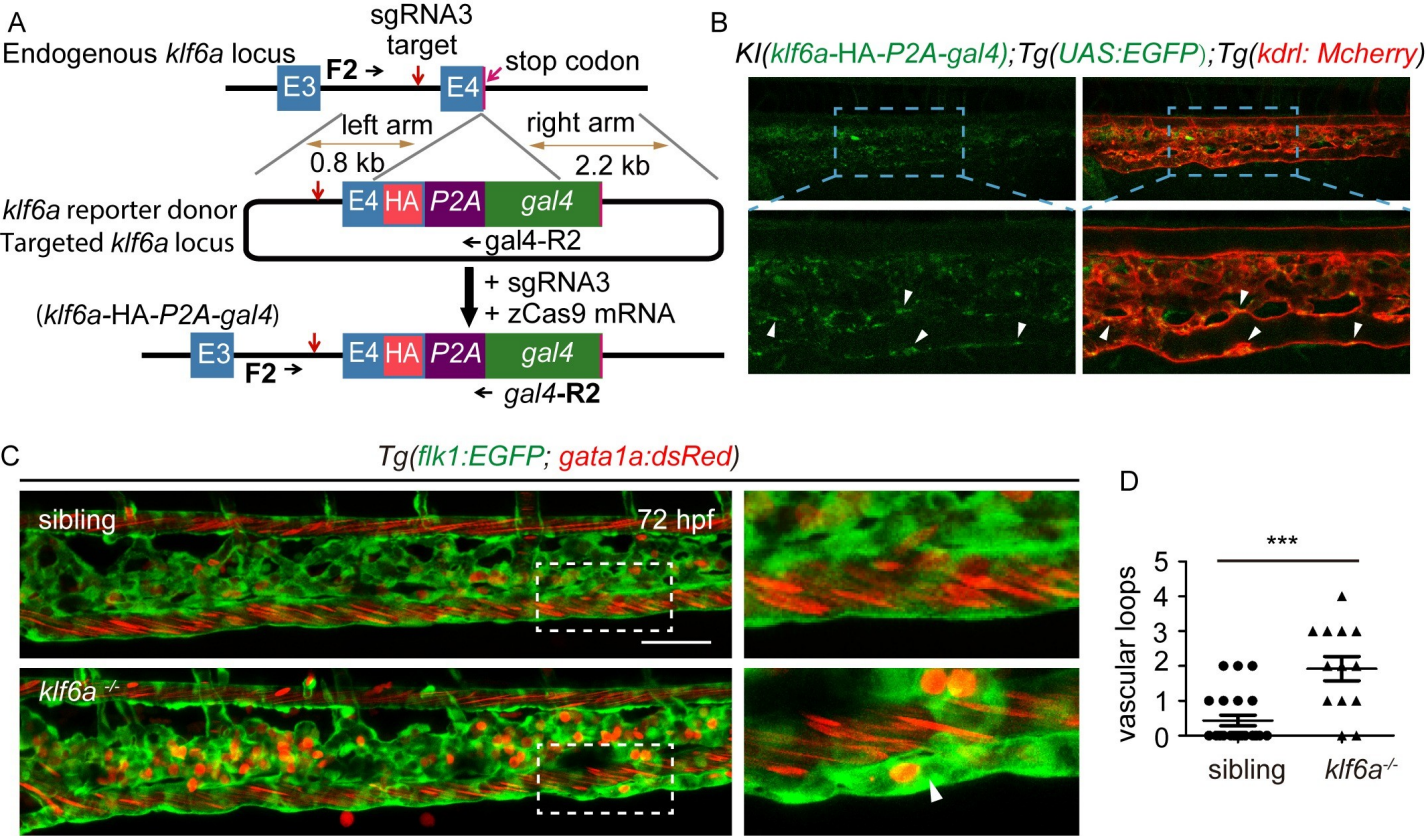
Transcription factor *klf6a* regulates CV pruning in zebrafish. (A) Generation of *KI (klf6a -*HA*-P2A-gal4)* fish. (B) Expression pattern of *KI(klf6a*-HA*-P2A-gal4)* fish. Arrowheads indicate the location of *klf6a* at the vasculature. (C) Role of *klf6a* in CV pruning in zebrafish embryos. Boxes show enlarged images of the CV. The arrowhead indicates the unpruned vessel. Scale bar: 50 μm. (D) Quantification of vascular loops in the sibling and *klf6a*^*-/-*^: sibling, *n* = 23 embryos; *klf6a*^*-/-*^, *n* = 13 embryos. *P* < 0.001. Student’s unpaired two-tailed t test.

To further validate whether *klf6a* is expressed in the CV of zebrafish embryos, we generated a KI fish of *KI(klf6a*-HA-*P2A-gal4);Tg(UAS*:*EGFP)* with CRISPR/Cas9 technology. An HA-*P2A-gal4* sequence was inserted into the last exon of the *klf6a* gene before the stop codon and fused into *klf6a*-HA-*P2A-gal4*, as described in Fig 3A. Then, the adult F_0_ *KI(klf6a-*HA*-P2A-gal4)* line was crossed with a *Tg(UAS*:*EGFP)* fish. We identified later generations by PCR analyses of their genomic DNA using target site-specific and donor-specific primers and subsequent sequencing analysis (S5B and S5C Fig). Consistent with the expression of *klf6a* in zebrafish in our WISH results and other reports [22,23], *KI(klf6a-*HA*-P2A-gal4)* embryos showed weak but clear expression of EGFP in the vasculature of the CV (Figs 3B and S5A).

These results reveal that *klf6a* is expressed in the CV of zebrafish embryos and is responsive to hemodynamic force *in vitro* and in zebrafish embryos.

### *Klf6a* regulates CV pruning in zebrafish embryos

To confirm the role of *klf6a* in zebrafish CV pruning, we injected *klf6a* MO into *Tg(flk1*:*EGFP;gata1a*:*dsRed)* embryos at the single-cell stage. We observed that knockdown of *klf6a* significantly increased the number of vascular loops in the CV at 72 hpf (average loops: control = 0.6667, *klf6a* MO = 1.7037; S6B and S6C Fig), although the embryos showed a normal morphology (S6A Fig). To further confirm the effect of *klf6a* on CV pruning in zebrafish embryos, we generated a zebrafish mutant of *klf6a* by CRISPR/Cas9, in which a 7 bp DNA fragment was deleted from exon 2 (S7 Fig). Compared to a sibling, ablation of *klf6a* increased the number of vascular loops in the CV at 72 hpf (average loops: sibling = 0.43478, *klf6*^*-/-*^ = 1.92308; Fig 3D). These results demonstrated that *klf6a* was required for CV pruning in zebrafish embryos.

### *Klf6a* regulates EC nucleus migration in CV pruning

**Fig 4:**
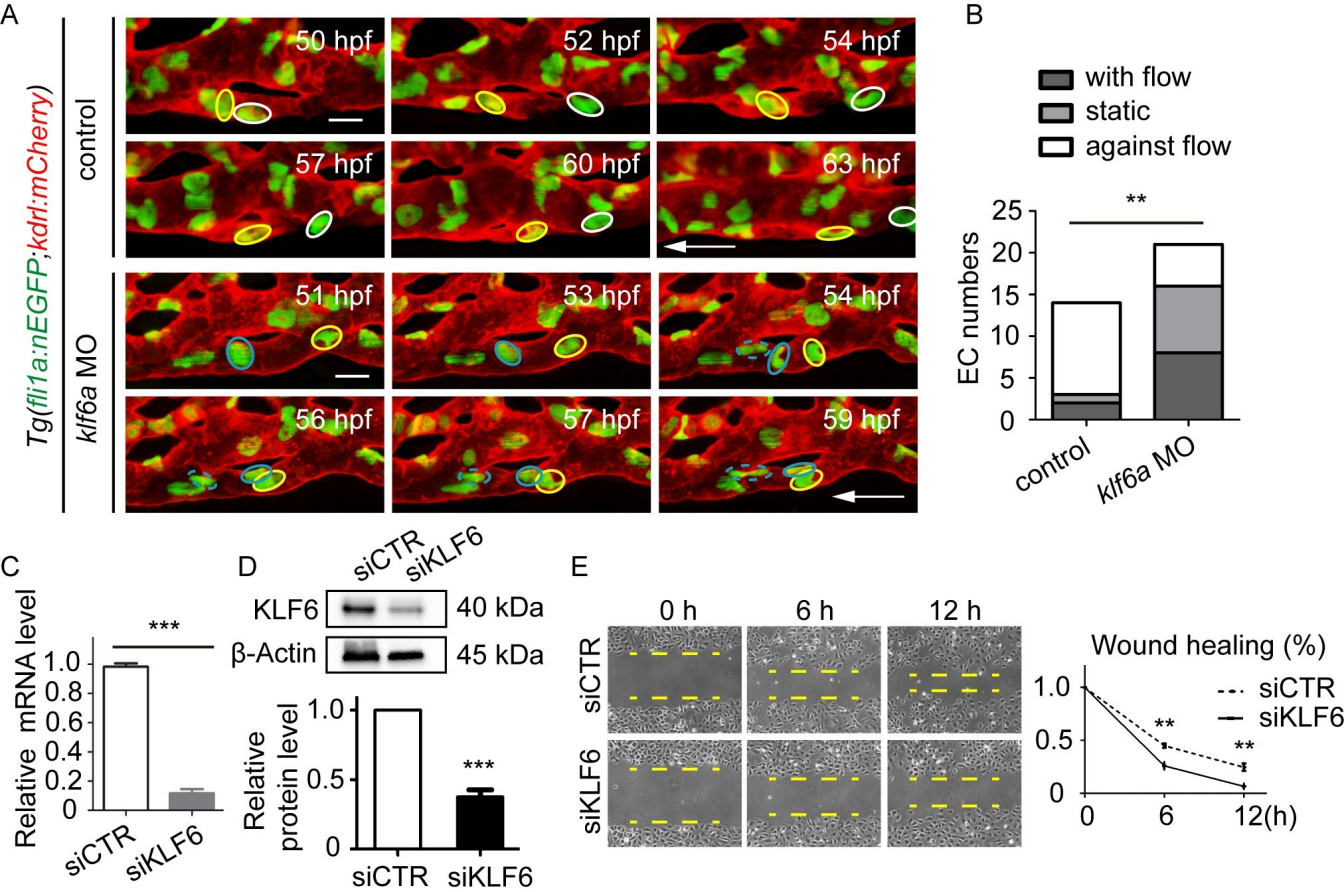
*Klf6a* regulates EC nucleus migration in CV pruning. (A) Time-lapse live imaging of *Tg(fli1a*:*nEGFP;kdrl*:*mCherry)* zebrafish embryos shows EC nucleus migration in CV pruning in control and *klf6a* morphant. The arrow shows the direction of the blood flow. Colored circles indicate EC nuclei. Scale bar: 10 μm. (B) The direction of EC migration within the regressing vessel. The direction is classified into three types: with the flow, static, and against the flow. A total of 10 vascular loops with 14 EC nuclei and 14 vascular loops with 21 EC nuclei were calculated in the control group and *klf6a* morphant group, respectively. *P* = 0.0058. Unpaired two-tailed chi-square test. (C) and (D) Efficiency of siKLF6 in human umbilical vein endothelial cells (HUVECs) at the mRNA (*P* = 0.00087) and protein (*P* = 0.0003) levels. (E) Wound healing of siCTR-transfected and siKLF6-transfected HUVECs: 6 h, *P* = 0.0013; 12 h, *P* = 0.0020. (C) to (E) Student’s unpaired two-tailed t test. *P < 0.05, **P < 0.01, ***P < 0.001.

To analyze the effect of *klf6a* on the migratory behavior of ECs in zebrafish CV pruning, we performed time-lapse live imaging of *Tg(fli1a*:*nEGFP;kdrl*:*mCherry)* transgenic embryos to trace the migration of the EC nucleus. As shown in Fig 4A, as the lower branch of the vascular loop in the control morphant became stenotic, the nuclei of two ECs (white and yellow circles) migrated against the blood flow from 50 to 54 hpf and from 54 to 60 hpf, respectively. However, in the *klf6a* morphant, the EC nucleus (blue circles) migrated against the blood flow from 51 to 56 hpf. The EC nucleus (shown in yellow) migrated in the direction of the blood flow. At 56 hpf, these two nuclei encountered each other, and they remained in the middle of the regressing vessel branch without significant migration from 56 to 59 hpf (Fig 4A).

Statistical analysis of our results revealed that 79% of nuclei (n = 11/14 ECs) migrated against the blood flow within the regressing branch in the control MO-injected embryos (Fig 4B). However, *klf6a* morphant showed caused a 54.8% decrease of nuclei (control: n = 11/14 ECs, *klf6a* MO: n = 5/21 ECs) migrating against the blood flow, a 23.8% increase of nuclei (control: n = 2/14 ECs, *klf6a* MO: n = 8/21 ECs) migrating with the blood flow, and a 31% increase of nuclei (control: n = 1/14 ECs, *klf6a* MO: n = 8/21 ECs) without migration (Fig 4B). Moreover, to evaluate the effect of KLF6 on EC migration *in vitro*, we transfected cultured HUVECs with siKLF6 and found that knockdown of KLF6 by siRNA significantly inhibited its expression at both the mRNA (Fig 4C) and protein (Fig 4D) levels. Next, we performed a wound healing assay to evaluate the effect of KLF6 on EC migration in HUVECs. The results revealed that knockdown of KLF6 by siRNA significantly inhibited EC migration in HUVECs (Fig 4E). These results reveal that *klf6a* regulates EC nuclei migration during vessel pruning.

Therefore, our results demonstrate that *klf6a* is required for the proper migration of EC nuclei in CV pruning in zebrafish embryos.

### *Klf6a* regulates junction remodeling and rearrangement of actin cytoskeleton in CV pruning

To further delineate the underlying mechanism of *klf6a* regulation of CV pruning in zebrafish embryos, we evaluated the effect of *klf6a* on junctions and actin cytoskeleton. We first checked the localization of KLF6 in cultured HUVECs *in vitro* by immunofluorescence staining with anti-KLF6 antibody and anti-VE-cadherin antibody (S8A Fig). We found that KLF6 was mainly located in the nuclei of HUVECs (S8A Fig). We then performed immunofluorescence staining to check the effect of KLF6 on actin cytoskeleton in cultured HUVECs using anti-VE-cadherin antibody and phalloidin. We observed actin filament bundles closely flanking VE-Cadherin-positive AJs in confluent siCTR-transfected HUVECs (S8B Fig). Knockdown of KLF6 by siKLF6 did not disrupt actin filament bundles closely flanking linear AJs, but caused excessive actin fiber stress (S8B Fig). To determine whether *klf6a* respond to blood flow to regulate EC rearrangement, we performed flow chamber experiment with HUVECs exposure to static (0 dyn/cm^2^) or FSS (12 dynes/cm^2^) for 24 hours. SiCTR-transfected HUVECs showed EC alignment in the direction of the flow in response to FSS. However, knockdown of KLF6 did not affect EC alignment in the direction of the flow, but caused excessive actin fiber stress (S8C Fig).

**Fig 5:**
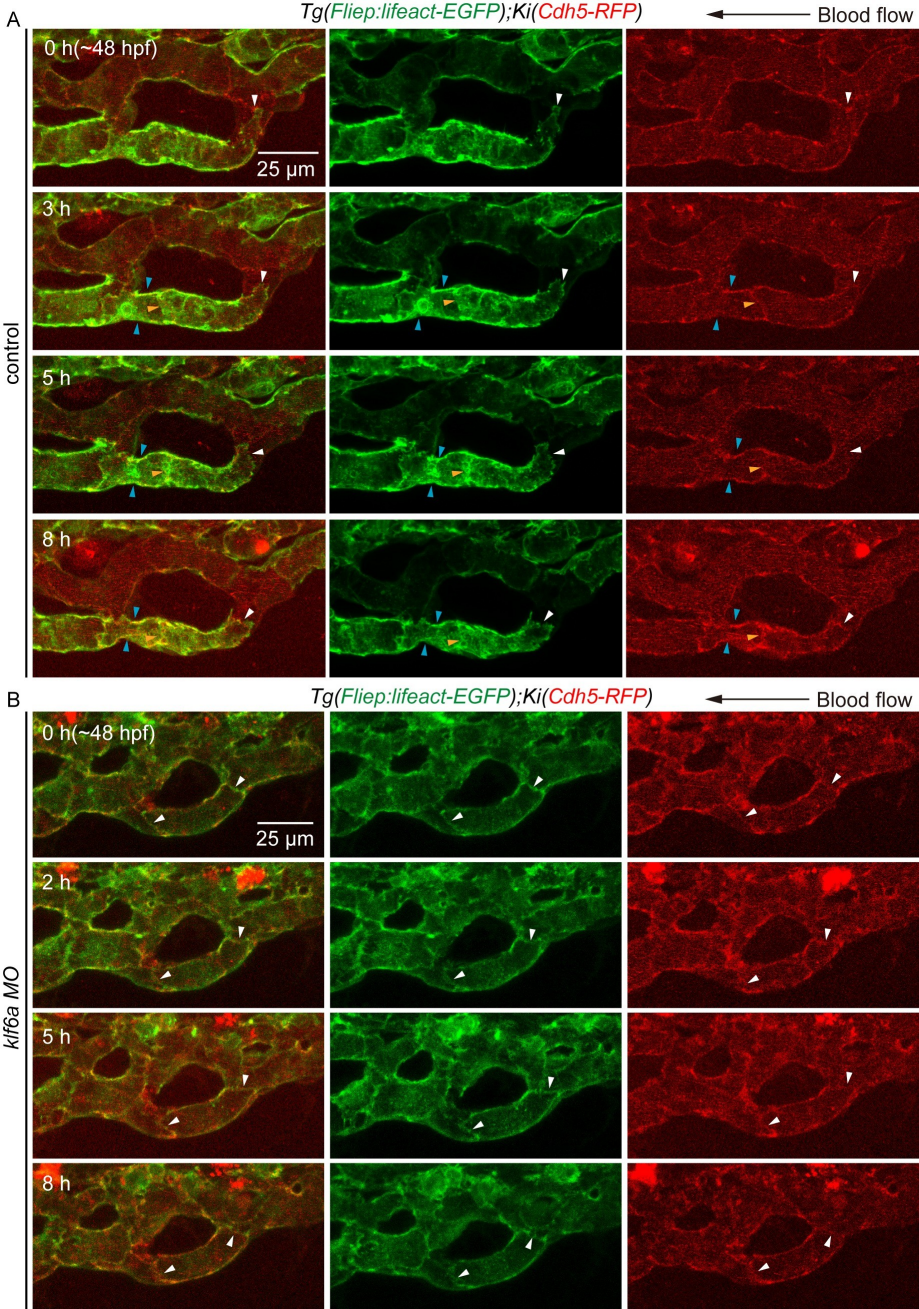
Knockdown of *klf6a* impairs junction remodeling and rearrangement of actin cytoskeleton in CV pruning. (A) and (B) Time-lapse live imaging of junction remodeling and actin cytoskeleton dynamics in CV pruning in *Tg(Fliep*:*Lifeact-EGFP);KI(cdh5-mRFP)* embryos. (A) Control embryo shows a retraction of junction (white arrowhead) and a shrinkage of its junctional ring (white and blue arrowheads) as multicellular tube became stenotic. F-actin forms at cdh5-positive junction (white, blue and yellow arrowheads), and undergoes a similar rearrangement as cdh5-positve junctions. 7 vascular loops are taken time-lapse live imaging. Scale bar: 25 μm. (B) Although *klf6a* morphant show F-actin co-localization with junction, however, neither junction nor F-actin does not go through remodeling (arrowhead). 5 vascular loops are taken time-lapse live imaging. Scale bar: 25 μm.

To further investigate the role of *klf6a* on actin cytoskeleton in vessel pruning *in vivo*, we followed a previous study to generate the transgenic line *Tg(Fliep*:*Lifeact-EGFP)* [24], in which the small F-actin-binding peptide Lifeact was fused with the EGFP protein and was driven by the endothelial *Fliep* promoter. To monitor junction remodeling and actin cytoskeleton dynamics, we performed time-lapse live imaging of the developing CV in *Tg(Fliep*:*Lifeact-EGFP)/KI(cdh5-RFP)* embryos at three stages: the multicellular tube stage, multicellular to unicellular tube transformation, and retraction. At the multicellular tube stage, we observed the synchronous retraction of junction and F-actin as vessel became stenotic in control embryos. As shown in Fig 5A, cdh5-positive AJ (white arrowhead) moved away from its adjacent junction on the right from 0 to 8 h, accompanied by the gradual shrinkage of junctional ring. F-actin co-localized with cdh5-positive AJ, and underwent a similar rearrangement as junction at the same time (white and blue arrowheads, Fig 5A). In addition, a gradual polymerization of F-actin can be observed at junction from 3 to 8 h (yellow arrowheads, Fig 5A). At the multicellular to unicellular tube transformation stage, we observed junction remodeling and F-actin polymerization/depolymerization as the branch became stenotic. As shown in S9A Fig, junction together with F-actin moved away to the right from 0 to 8 h (white arrowhead). In the non-narrow region, F-actin depolymerized from 2 to 5 h (white arrow), whereas F-actin polymerized at junctions in the narrow region (yellow arrowhead). During retraction, the dissociation and retraction of F-actin was clearly visible (S9B Fig). However, *klf6a* morphants showed compromised junction remodeling and rearrangement of actin cytoskeleton, which resulted in defective branch stenosis during CV pruning (Fig 5B). As shown in Fig 5B, knockdown of *klf6a* did not lead to movement or retraction of junction nor the rearrangement of actin rearrangement from 0 to 8 h, although F-actin co-localized with cdh5-positive AJ (white arrow, Fig 5B).

Taken together, these results show that junction remodeling and actin cytoskeleton dynamics are involved in CV pruning in zebrafish and that *klf6a* regulates both processes.

### *Tagln2* functions as a direct downstream target of *klf6a* in zebrafish embryos

**Fig 6:**
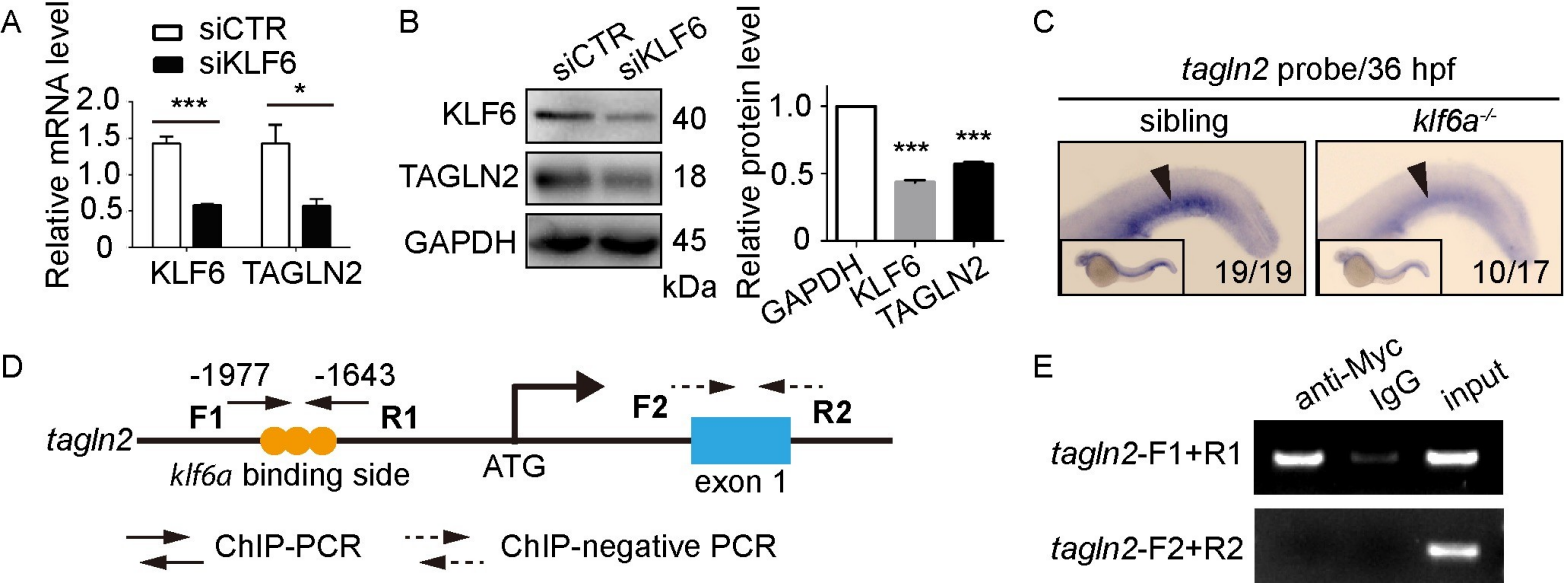
*Tagln2* is a direct downstream target of *klf6a*. (A) and (B) SiKLF6 in HUVECs efficiently decrease TAGLN2 expression at the mRNA (KLF6: *P* = 0.0009, TAGLN2: *P* = 0.0352) and protein (KLF6: *P* < 0.0001, TAGLN2: *P* < 0.0001) levels. Student’s unpaired two-tailed t test. *P < 0.05, ***P < 0.001. (C) WISH of the *tagln2* gene of sibling and *klf6a*^*-/-*^ zebrafish at 36 hpf. Arrowheads indicate the CVP region expressing *tagln2*. (D) Structure of the *tagln2* promoter. The primer *tagln2*-F1+R1 contains the conserved Klf6a binding side-CACCC, whereas *tagln2*-F2+R2 is a negative control located in the *tagln2* exon 1. (E) ChIP-PCR of the *tagln2* promoter shows that Klf6a can bind directly to the *tagln2* promoter.

The influence of transcription factor *klf6a* on actin cytoskeleton arrangement raised the possibility that cytoskeleton-associated genes could be involved in *klf6a*-mediated CV pruning in zebrafish embryos. After analyzing the available RNAseq profiles of Klf6-related transcripts in non-EC types reported by Laitman *et al*. [25], we found that two actin-related genes were downregulated by Klf6: activity-regulated cytoskeleton-associated protein (Arc) and transgelin 2 (Tagln2). *Tagln2* expression is enriched in the vasculature of zebrafish embryos [26]. Therefore, we speculated that *tagln2* could be the missing downstream target of *klf6a* for regulating CV pruning in zebrafish embryos. In support of this assumption, we found that knockdown of KLF6 by siKLF6 decreased TAGLN2 expression at the mRNA and protein levels *in vitro* (Fig 6A and 6B). In addition, the *klf6a* homozygous mutant showed decreased mRNA for *tagln2* in the CVP region at 36 hpf, as determined by WISH (Fig 6C).

To confirm whether *tagln2* is a direct target of *klf6a* in zebrafish, we performed a bioinformatics analysis to predict the potential sequence within the zebrafish *tagln2* promoter to which transcription factor Klf6a can bind. As shown in Fig 6D, three CACCC sequences to which Klf6a can bind are located in the -1977 to -1643 bp region of the *tagln2* promoter. We then performed chromatin immunoprecipitation (ChIP) assay on the zebrafish embryos to validate this. The results revealed that Klf6a can bind to this promoter region within the *tagln2* gene but not its exon 1 (Fig 6E).

These results demonstrate that *tagln2* is the direct downstream target of *klf6a* in zebrafish, which suggests that *tagln2* could be involved in *klf6a*-mediated CV pruning.

### *Tagln2* regulates CV pruning in zebrafish by promoting EC nucleus migration

**Fig 7:**
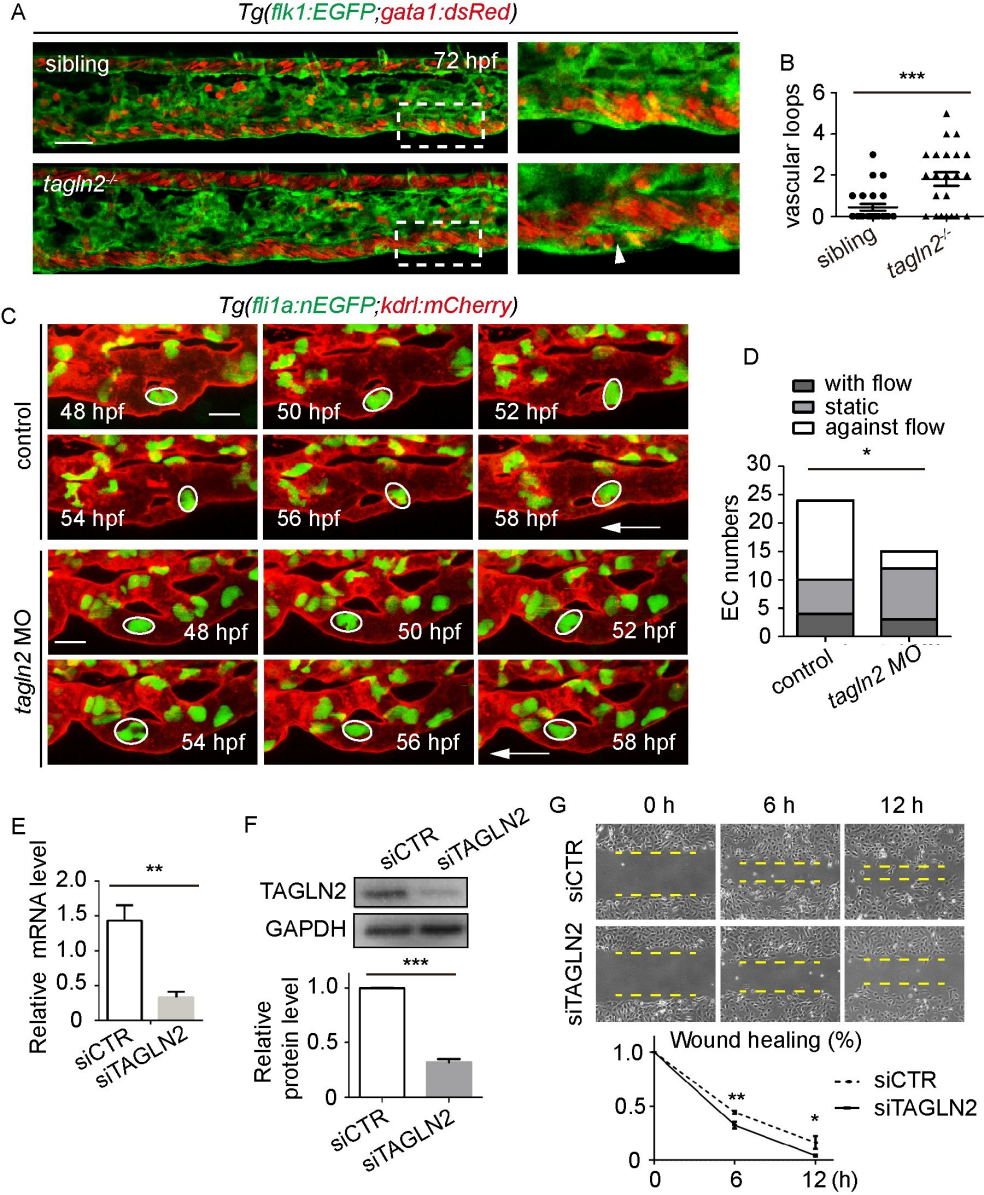
*Tagln2* regulates CV pruning by promoting EC nucleus migration. (A) Role of *tagln2* in CV pruning in zebrafish embryos. Boxes show enlarged images of the CV. The arrowhead indicates unpruned vessel. Scale bar: 50 μm. (B) Quantification of vascular loops in sibling and *tagln2*^*-/-*^ embryos: sibling, n = 25 embryos; *tagln2*^*-/-*^, n = 23 embryos. *P* = 0.0004. Student’s unpaired two-tailed t test. (C) Time-lapse live imaging of *Tg(fli1a*:*EGFP;kdrl*:*mCherry)* embryos shows EC nucleus migration in CV pruning in control or *tagln2* morphant. Arrows indicate the direction of the blood flow. Colored circles indicate the EC nuclei. Scale bar: 10 μm. (D) Direction of EC nucleus migration in the regressing vessel. The direction is classified into three types: with the flow, static, and against the flow. A total of 12 vascular loops with 24 EC nuclei and 8 vascular loops with 15 EC nuclei were calculated in the control group and *tagln2* morphant, respectively. Unpaired two-tailed chi-square test. *P* = 0.0471. (E) and (F) Efficiency of siTAGLN2 in HUVECs at the mRNA (*P* = 0.0094) and protein (*P* < 0.0001) levels. (G) Wound healing of siCTR-transfected and siTAGLN2-transfected HUVECs. Statistics for wound healing after siCTR and siTAGLN2 transfection: 6 h, *P* = 0.0037; 12 h, *P* = 0.0252. (E)-(G) Student’s unpaired two-tailed t test. *P < 0.05, **P < 0.01, ***P < 0.001.

To determine the role of *tagln2* in zebrafish CV pruning, we first validated the efficiency of *tagln2* MO using a tagln2-EGFP fusion protein (S10A Fig), the expression of which was clearly inhibited by *tagln2* MO. This MO was then used to knockdown the *tagln2* gene in embryos to evaluate the effect on CV pruning at 72 hpf (S10B and S10C Fig). Statistical analysis clearly showed that the disruption of *tagln2* by MO increased the number of vascular loops in the CV at 72 hpf (average loops: control = 0.6, *tagln2* MO = 1.4615; S10C and S10D Fig). To further validate the role of *tagln2* in CV pruning, we then generated the *tagln2* mutant in zebrafish using CRISPR/cas9 technology, in which a 7 bp fragment was deleted from exon 1 (S11 Fig). Compared to the WT sibling, *tagln2* homozygous mutations showed a significant increase in the number of vascular loops at the CV at 72 hpf (average loops: sibiling = 0.44, *tagln2*^*-/-*^ = 1.81818; Fig 7A and 7B).

Next, we analyzed the effect of *tagln2* on EC behavior using time-lapse live imaging of *Tg(fli1a*:*nEGFP;kdrl*:*mCherry)* embryos. In control MO-injected embryos, EC nucleus migration against the blood flow (white circle) could be readily observed within regressing vessel branches from 48 to 54 hpf (Fig 7C). However, in the *tagln2* morphant, EC nuclei (white circle) showed no obvious movement from 48 to 58 hpf (Fig 7C). Knockdown of *tagln2* by MO caused a 38% decrease in EC nuclei (control: n = 14/24 ECs, *tagln2* MO: n = 3/15 ECs) migration against the blood flow, a 3% increase in EC nuclei (control: n = 4/24 ECs, *tagln2* MO: n = 3/15 ECs) migration with the blood flow, and a 35% increase in ECs nuclei (control: n = 6/24 ECs, *tagln2* MO: n = 9/15 ECs) without migration (Fig 7D). These results strongly support our proposition that *tagln2* is required for CV pruning by regulating EC nucleus migration. To evaluate the effect of TAGLN2 on EC migration *in vitro*, we used siTAGLN2 to silence TAGLN2 in cultured HUVECs and found that TAGLN2 expression was significantly inhibited at both the mRNA (Fig 7E) and protein (Fig 7D) levels. The results of a wound healing assay performed to evaluate the effect of TAGLN2 on EC migration in HUVECs revealed that knockdown of TAGLN2 by siRNA significantly inhibited EC migration in HUVECs (Fig 7G).

Taken together, our results demonstrated that *tagln2* acts as a downstream target of *klf6a* and regulates CV pruning in zebrafish by promoting EC nucleus migration.

### *Tagln2* regulates junction remodeling and the arrangement of actin cytoskeleton in CV pruning

To further understand the role of *tagln2* in actin cytoskeleton, we performed immunofluorescence staining of confluent HUVECs *in vitro* with anti-TAGLN2 and found that TAGLN2 co-localized with F-actin at a significant level (S12A Fig). This indicated a potential role of TAGLN2 in actin cytoskeleton arrangement. To evaluate the effect of TAGLN2 on actin cytoskeleton *in vitro*, we then performed an immunofluorescence assay on cultured HUVECs with anti-VE-cadherin antibody and phalloidin. We observed actin filament bundles closely flanking VE-cadherin-positive AJs in confluent siCTR-transfected HUVECs (S12B Fig). Knockdown of TAGLN2 by siTAGLN2 did not disrupt actin filament bundles closely flanking linear AJs but caused excessive actin fiber stress (S12B Fig).

**Fig 8:**
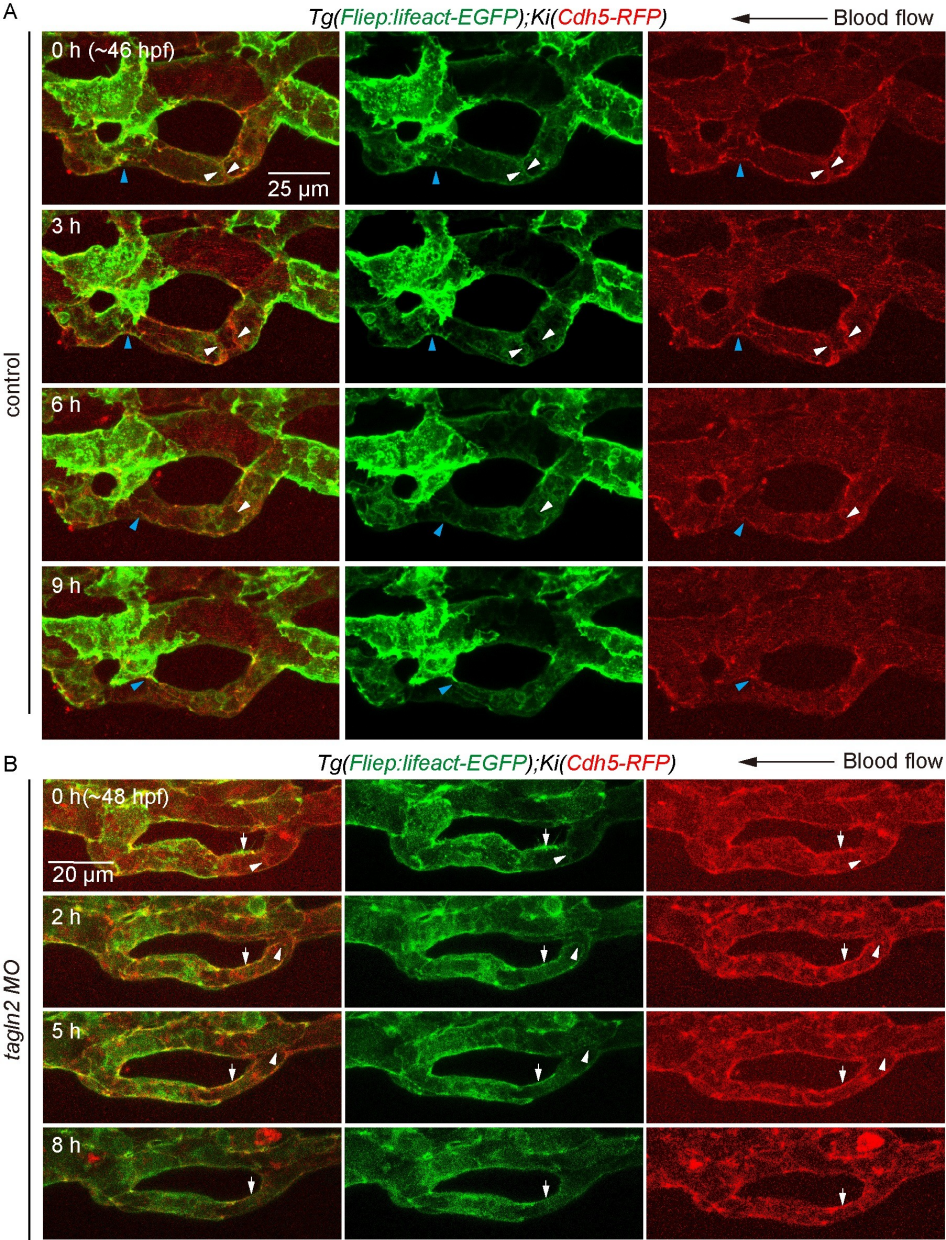
Knockdown of *tagln2* impairs junction remodeling and rearrangement of F-actin cytoskeleton in CV pruning. (A) and (B) Time-lapse live imaging of junction remodeling and actin cytoskeleton dynamics in CV prunig in *Tg(Fliep*:*Lifeact-EGFP);KI(cdh5-mRFP)* embryos. (A) Control embryo shows a detachment of junction (blue arrowheads) and an opposite direction of junction movement (white arrowheads) as multicellular tube became stenosis. F-actin forms at cdh5-positive junction (white and blue arrowheads), and undergoes a similar rearrangement as cdh5-positve junctions. Seven vascular loops are taken time-lapse live imaging at the stage of multicellular tube. Scale bar: 25 μm. (B) In *tagln2* morphant, cdh5-positive junction moves to the right and connected with the adjacent one (white arrowheads), and F-actin undergoes similar rearrangement within it (white arrowheads). However, depolymerization of F-actin is also observed at junctions (white arrowheads). Six vascular loops are taken time-lapse live imaging. Scale bar: 20 μm.

To further investigate the role of *tagln2* on actin cytoskeleton in vessel pruning *in vivo*, we performed time-lapse live imaging of the developing CV in *Tg(Fliep*:*Lifeact-EGFP)/KI(cdh5-RFP)* embryos. We found that knockdown of *tagln2* by *tagln2*-MO significantly inhibited junction remodeling and actin cytoskeleton dynamics in zebrafish CV pruning (Fig 8). As shown in Fig 8A, control embryo showed a gradual detachment of junctions (blue arrowheads) from 0 to 9 h, and exhibited an opposite direction of junction movement (white arrowheads) from 0 to 3 h as multicellular tube became stenotic. F-actin formed at junction, and underwent similar rearrangement as junction remodeling (white and blue arrowheads, Fig 8A). However, knockdown of *tagln2* resulted in compromised junction remodeling and actin cytoskeleton dynamics during CV pruning (Fig 8B). In *tagln2* morphants, the junction moved to the right of adjacent junction, and the two junctions came into contact with each other from 0 to 5 h (white arrowhead). However, F-actin located at junction, we can observe a depolymerization of F-actin at the other junction from 0 to 8 h (white arrows).

Taken together, these findings show that *tagln2* regulates junction remodeling and rearrangement of actin cytoskeleton in vessel pruning, thereby promoting CV formation in zebrafish embryos.

## Discussion

Vessel pruning has been well described in different vascular beds of zebrafish, such as the hindbrain vasculature [4], CrDI in the eye [5], SIVs [6], and vISVs [2]. In this study, compelling evidence from our live imaging showed that the large-diameter vessel of the CV is pruned from ventral capillaries of the CVP in zebrafish embryos characterized by vessel stabilization/regression and EC rearrangement within the regressing branch.

Vessel pruning, which is essential for the creation of an ordered and hierarchical vascular network, eventually results in a ramified vasculature [1,7]. Flow-dependent endothelial rearrangement drives vessel pruning in a mouse retina model [2] and in various vascular beds of zebrafish embryos [27]. However, it is unclear how EC rearrangement causes vessel stenosis within the regressing vessel. By tracing EC behavior with time-lapse imaging of *Tg(fli1a*:*nEGFP;kdrl*:*mCherry)* zebrafish embryos, we found that EC nucleus migration against the direction of the blood flow clearly precedes vessel stenosis (Fig 2B). Moreover, we observed junction remodeling and actin cytoskeleton dynamics as vessel stenosis in *Tg(Fliep*:*Lifeact-EGFP)/KI(cdh5-RFP)* embryos (Figs 5A, 8A and S9). These results indicate that both EC nucleus migration, junction remodeling and actin cytoskeleton dynamics contribute to vessel stenosis during CV pruning in zebrafish embryos.

Several groups of researchers have performed imaging of blood flow and vascular remodeling in different vascular beds and analyzed the relationship between them. Reductions in blood flow inhibit vessel pruning [4,5,10,11]. However, the precise role of blood flow in vessel pruning remains unclear. By tracing EC behavior with time-lapse imaging of *Tg(fli1a*:*EGFP;gata1*:*dsRed)* embryos, we found that vessel stenosis precedes no perfusion in the regressing vessel (Fig 2C). Further, the graded difference in blood flow between the two branches increases as one of the branches becomes stenotic (Fig 2C–2E). Reduced blood flow in embryos treated with tricaine and *tnnt2a* MO resulted in compromised CV pruning (S3 Fig). Differences in pressure between branches can be used to predict the location of sprouting, in which sprouts migrate form from vessels at lower pressure toward vessels at higher pressure [11]. In future studies, it could be useful to predict which branch is stable or pruned, based on flow dynamics.

Actin cytoskeleton plays important roles in angiogenic sprouting [28], aortic lumen expansion [29], and flow-induced vascular dilation and remodeling [30]. In addition, FSS induces actin cytoskeleton remodeling *in vitro* [31]. Our time-lapse live imaging of *Tg(Fliep*:*Lifeact-EGFP)/KI(cdh5-RFP)* embryos showed junctional detachment and retraction in vessel pruning (Figs 5A, 8A and S9), which resembles anastomosis in reverse [32]. In anastomosis of DLAVs, F-actin forms at junctions [24]. In our study, we found that F-actin forms at junction, and go through similar rearrangement as junction remodeling during CV pruning. Furthermore, F-actin depolymerization was found in the non-narrow region at multicellular to unicellular tube transformation stage, whereas F-actin polymerization at junctions seems to be preferred in the narrow region as branch stenosis (S9 Fig). These results suggest that actin cytoskeleton is essential for flow-induced vessel pruning.

Our strong results support the important role of *klf6a* in CV pruning in zebrafish embryos. First, results from WISH (S4 Fig) and the *KI(klf6a-*HA*-P2A-gal4)* fish (Figs 3B and S5A) clearly confirm that *klf6a* is expressed in the ECs of the CV in zebrafish embryos, consistent with a previous report [23]. Second, *klf6a* is responsive to hemodynamic force *in vitro* and *in vivo* (S4C and S4D Fig). Third, a disruption of *klf6a* function impairs EC nucleus migration against the blood flow within the regressing branch (Fig 4A and 4B) and results in compromised CV pruning (Figs 3C, 3D and S6). Furthermore, *klf6a* regulates junction remodeling and actin cytoskeleton dynamics *in vivo* (Fig 5).

Researchers previously identified a crucial role of TAGLN2 in regulating actin cytoskeleton assembly [33–35]. Actin-binding protein *tagln2* functions as a direct downstream target of *klf6a* to regulate CV pruning in zebrafish (Figs 6 and 7). First, *klf6a* regulates *tagln2* expression in the zebrafish CVP region and *in vitro* (Fig 6A and 6C). Second, Klf6a can bind to the *tagln2* promoter (Fig 6D and 6E). Third, the *tagln2* morphant and mutants show defective CV pruning (Figs 7A, 7B and S10). Moreover, our results show that a disruption in *tagln2* results in defective EC nucleus migration, junction remodeling, and actin cytoskeleton dynamics *in vivo* (Figs 7C, 7D and 8). These results mimic the phenotype observed in the *klf6a* morphant or mutants.

In conclusion, our results demonstrate that the *klf6a-tagln2* axis promotes vessel pruning in CV formation in zebrafish by regulating EC rearrangement and actin cytoskeleton dynamics.

## Materials and methods

### Ethics statement

All zebrafish experimentation was approved by the Ethics Committee of Chongqing University Affiliated Cancer Hospital. The ethical review number: CZLS2021172-A.

### Zebrafish breeding

Zebrafish (*Danio rerio*) embryos were raised as described previously [36]. All zebrafish maintenance and experiments were carried out in accordance with guidelines approved by the Ethics Committee of Chongqing University. The following transgenic fish lines were used: *Tg(flk1*:*GFP)* [37], *Tg(kdrl*:*mCherry)* [38], *Tg(fli1*:*nEGFP)* [39], *Tg(gata1a*:*dsRed)* [40], *Tg(fli1a*:*B4GALT1-mCherry)* [15], and *Tg(Fliep*:*Lifeact-EGFP)*, *Tg(UAS*:*EGFP)* [24].

*Tg(fli1a*:*B4GALT1-mCherry)* zebrafish was generated as described previously [15]. Briefly, embryos at the single-cell stage were co-injected with pTol fli1a:B4GALT1-mCherry plasmid (25 ng) and Tol2 transposase mRNA (25 ng). Embryos showing red fluorescence were selected and grown to adulthood. The founder was identified by specific expression of B4GALT1-mCherry in the blood vessels of their progeny.

*Tg(Fliep*:*Lifeact-EGFP)* zebrafish was generated as described previously [24]. Briefly, embryos at the single-cell stage were co-injected with pTol fliep:Lifeact-EGFP plasmid (25 ng) and Tol2 transposase mRNA (25 ng). Embryos showing green fluorescence were selected and grown to adulthood. The founder was identified by specific expression of Lifeact-EGFP in the blood vessels of their progeny.

### Microinjection of MO oligonucleotides

All MO were ordered from Gene Tools LIC. 4 ng antisense oligonucleotide (MO) was injected into embryos at the single-cell stage. The MO sequences are listed in S1 Table. The efficiency of the *tagln2* MO was validated by the pcDNA3.1-tagln2-EGFP plasmid, which consists of the sequence complementary to the *tagln2* MO.

### Generation of the zebrafish *klf6a/tagln2* mutant and KI by CRISPR/Cas9

The generation of zebrafish mutants and KI was performed with CRISPR/Cas9 by Nanjing XinJia Medical Technology (China). Briefly, the *klf6a* short guide RNA (sgRNA) was targeted to exon 2, and the target sequence was GAATTCGGATGCCAGCAGCGAGG. The *tagln2* sgRNA was targeted to exon 1, and the target sequence was CACCTCGCGACTCAGACCGTAGG. SgRNA was synthesized following the manufacturer’s protocol (NEB, E2050s). Cas9 mRNA was synthesized with Ambion mMESSAGE mMACHINE mRNA transcription synthesis kits. Cas9 mRNA (600 ng/μL) and *klf6a* gRNA or *tagln2* gRNA (300 ng/μL) were co-injected into WT embryos at the single-cell stage. The embryos were raised to adulthood, and the founder (F_0_) mutants were identified by genomic PCR, followed by sequencing. To confirm germline-transmitted mutations of both the *klf6a* and *tagln2* genes, we outcrossed adult F_0_ founders with WT AB fish to obtain heterozygous mutant zebrafish (F_1_). Adult F_1_ mutants were validated by genomic PCR and sequencing. The same heterozygous mutants of F_1_ were in-crossed to generate homozygous mutant embryos (F_2_) and used for further experimental analyses. The primers used to genotype the *klf6a* and *tagln2* mutants are listed in S2 Table.

The KI sgRNA was targeted to the last exon of the zebrafish *klf6a*. The *klf6a* reporter donor plasmid *klf6a-*HA*-P2A-gal4* consists of three parts: a left arm, a *klf6a-*HA*-P2A-gal4* coding sequence, and a right arm. The left arm goes from the 5′ side of the sgRNA to the end of exon 4. The right arm includes the stop codon and 3′ regulatory elements of *klf6a*. The sgRNA, cas9 mRNA, and donor plasmid were co-injected into zebrafish embryos at the single-cell stage. The embryos were raised to adulthood, and the founder (F_0_) transgenic lines were identified by genomic PCR, followed by sequencing. To confirm the germline-transmitted transgenic line, adult F_0_ founders were outcrossed with *Tg(UAS*:*EGFP)* fish to obtain the F_1_ transgenic line, followed by identification with genomic PCR and green fluorescence. The F_1_ transgenic line were outcrossed to generate F_2_ embryos for further experimental analyses. Target side-primer (F2) and donor-primers- (gal4-R2) were used in PCR to identify the successful KI zebrafish. The primers used to identify *KI(klf6a-*HA*-P2A-gal4)* are listed in S2 Table.

To generate a KI fish of *KI(Cdh5-mRFP)* line, the *mRFP* sequence is inserted into the last exon (exon 12) of zebrafish *cdh5* gene before the stop code and fused into *cdh5-mRFP*. Briefly, cdh5-sgRNA, cas9 mRNA and the donor plasmid were co-injected into zebrafish embryos at the single-cell stage. The embryos were raised to adulthood, and the founder (F_0_) transgenic lines were identified by genomic PCR of target site-primer (chd5-F4) and donor-specific primer (mRFP-5-R) and subsequent analysis of sequencing. The primers used to identify *KI(cdh5-mRFP)* are listed in S2 Table.

### Tricaine treatment

Tricaine was dissolved in ddH_2_O at a concentration of 4 mg/mL, and the pH was adjusted to 7.2 ± 0.2 for storage. Tricaine was added into the egg water to a final concentration of 0.06 mg/mL at 30 hpf until imaged at 72 hpf.

### Time-lapse live imaging of zebrafish embryos

All confocal imaging was performed with a Leica SP8 confocal microscope at 28.5°C. Time-lapse live imaging of zebrafish embryos was performed as described previously [36]. Briefly, to prevent the formation of pigment, embryos were treated with 0.003% 1-phenyl-2-thiourea (PTU; Sigma) at 24 hpf. For time-lapse live imaging, the zebrafish embryos were immobilized with 1% low-melt agarose (Sigma) without tricaine treatment. Stacks were taken every 2 or 2.5 h with a step size of 1.5 μm.

### Calculation of the velocity of RBCs

The velocity of the blood flow was calculated as described previously [41]. Briefly, a Leica SP8 high-speed module was used to record videos of RBCs with the setting 512 × 200 (one stack was completed in 29 ms). We calculated the blood flow velocity in the CV manually using ImageJ as follows: For each video, we calculated the movement of three or four different RBCs, taking systole and diastole into account. Only clear and stable RBC movements in the CV area were converted from video into images and analyzed with ImageJ. We converted pixel units into known distance (pixels/um) using the scale bar provided by LAS X and ImageJ for conversion. The travel distance of single RBC was calculated as displacement distance (um) as a function of time (ms). The average blood flow velocity was calculated from the displacement and the time data.

### WISH

WISH of zebrafish embryos was performed as described previously [41]. Images were captured by microscope (Zeiss Stemi 2000-C). The probe primers are listed in S2 Table.

### ChIP assay

ChIP assay was performed with a Cell Signal Technology (S9004) kit instruction book. As there is no specific antibody of Klf6a in zebrafish for the ChIP assay, we used the Klf6a overexpression system, consistent with a previous report [23]. *Klf6a-myc* mRNA was injected into zebrafish embryos at the single-cell stage, and then the embryos were collected for ChIP assay at 36 hpf. The klf6a-myc plasmid was a gift from Professor Feng Liu at the Institute of Zoology, CAS of China. The Myc antibody was used to IP the sequence of *tagln2* targeting by Klf6a. IgG was used as a negative control. The eluted DNA (precipitated by the Myc or IgG antibody) was assayed by PCR. The primers were designed according to the klf6a-CACCC binding sites. Nonspecific primers were used as negative controls. The primers are summarized in S2 Table.

### Cell culture, immunofluorescence staining, and Western blotting

Primary HUVECs were obtained from Science Research Laboratories and cultured with EC medium (ECM) and 10% fetal bovine serum (FBS). All antibodies used in this study are listed in S3 Table. KLF6 siRNA is ordered from Santa Cruz (sc-38021). The Tagln2 siRNA sequence is 5′-CUCUGUGUCCUCCGUUCAUTT-3′ and 5′-AUGAACGGAGGACACAGAGTT-3′ (Shanghai GenePharma). Immunofluorescence staining and western blotting were performed as described previously [41].

### Quantitative RT-PCR analysis

Quantitative real-time PCR (qPCR) was performed with TB Green Fast qPCR Mix (RR430A; Takara). The qPCR primers are listed in S2 Table.

### Flow chamber experiment

The flow chamber experiment was performed as described previously [41]. A custom-built flow chamber consisting of two parallel plates made of polymethyl-methacrylate was used to apply uniform shear stress on HUVEC monolayers. The lower plate was flat, and a rectangular channel was engraved on the upper plate with an automatic milling machine. HUVECs were grown to confluence on a patch of polylysine slide coated with fibronectin. The patch was then placed on the lower plate, and the upper plate was mounted to form a sealed channel of parallel-plate geometry.

The actual shear stress (t) applied to the cells can be expressed in terms of volumetric flow rate (*Q*), medium viscosity (*μ*; 1.3 × 10^2^ Pa ▪ s), and width (*w*; 25 mm) and height (*h*; 0.25 mm) of the channel: τ = *Qμ*/*wh*^2^. In our setup, flow rates of 15 mL/min were applied to obtain shear stress values of 12 dyn/cm^2^. The flow rate was controlled with a peristaltic roller pump.

### Statistical analyses

To quantify defective CV formation, we quantified seven segments of each embryo. All statistical analyses were done with GraphPad Prism 5. The statistical significance of the difference between control and experimental groups was determined with Student’s unpaired two-tailed t test or unpaired two-tailed chi-square test. Data are presented as means ± SEM. *P* > 0.05 was considered non-significant. *P < 0.05, **P < 0.01, and ***P < 0.001, as shown in figures and figure legends.

## Supporting information

S1 Fig EC rearrangement in CV pruning in zebrafish. (A) Schematic diagram of *KI(cdh5-mRFP)* fish. (B) Sequence of *KI(cdh5-mRFP)* fish. (C) Identification of *KI(cdh5-mRFP)* by PCR analysis. (D) Time-lapse live imaging of *Tg(fli1a*:*EGFP);KI(cdh5-mRFP)* embryos shows EC rearrangement in CV pruning. The arrow shows the direction of the blood flow. Colored circles indicate the EC nuclei. Six time-lapse live imaging were taken. Scale bar: 10 μm.
(TIF)

S2 Fig There is no polarity against the blood flow during CV pruning. (A) Imaging of *Tg(B4GALT1-mCherry; fli1a*:*nEGFP)* embryos shows EC polarity against blood flow in intersegmental vessels (ISVs) at 48 hpf. The green indicates nucleus, the red indicates Golgi. Box indicates the enlarged image of Arterial ISV (aISV). The arrow indicates the direction of the blood flow. Scale bar: 75 μm. (B) EC polarity is not involved in CV pruning during EC migration. The arrow indicates the direction of the blood flow. Nine moves were analyzed. among 22 venous ECs, 19 ECs did not reveal polarities against the blood flow. Scale bar: 25 μm.
(TIF)

S3 Fig The decrease in blood flow disrupts CV pruning. (A) and (C) The representative images of gross morphology in bright field at 72 hpf. (B) and (D) The representative image of CV pruning in zebrafish, and images were taken at 72 hpf. Boxes indicate the enlarged image of CV. Arrowheads indicate unpruned vessel. Scale bar: 50 μm. (A) and (B) Embryos treated with ddH_2_O or 0.06 mg/ml tricaine from 30 hpf to 72 hpf. (C) and (D) Embryos injected with 1ng control MO or 0.2 ng *tnnt2a* MO. (E) The RBC velocity in the control group and the group treated with tricaine. Six videos (4 RBCs/videos) and nine videos (4 RBCs/ video) were used to calculate RBC velocity in the control and tricaine treatment groups, respectively. *P* < 0.0001. (F) Quantification of vascular loops in the CV. Control: *n* = 23 embryos, tricaine treatment: *n* = 28 embryos. *P* = 0.0004. (G) The RBC velocity in the control group and the *tnnt2a* MO-injected group. 6 videos (3 RBCs/video) and 10 videos (4 RBCs/ video) were used to calculate RBC velocity in the control and *tnnt2a* morphant groups, respectively. *P* < 0.0001. (H) Quantification of vascular loops in the CV. Control: *n* = 24 embryos, *tnnt2a* morphant: *n* = 39 embryos. *P* = 0.0132. Student’s unpaired two-tailed t test. *P < 0.05, **P < 0.01, ***P < 0.001.
(TIF)

S4 Fig *Klf6a* responds to flow shear stress. (A) WISH of the *klf2a*, *klf2b*, *klf4*, *klf6a*, and *klf6b* genes of zebrafish at 36 hpf. The arrowhead indicates a CVP with *klf6a* expression. (B) and (C) Relative mRNA (*P* < 0.0001) and protein (*P* = 0.0010) levels of KLF6 after treatment with 0 or 12 dyn/cm^2^ flow shear stress (FSS). Student’s unpaired two-tailed t test. **P < 0.01, ***P < 0.001. (D) WISH of the *klf6a* gene in zebrafish at 36 hpf after treatment with tricaine or *tnnt2a* MO. Arrowheads indicate the CVP region. (E) Quantification of *klf6a* relative mRNA level in zebrafish embryos at 36 hpf after treatment with tricaine (*P* = 0.0411) or *tnnt2a* MO (*P* = 0.0317).
(TIF)

S5 Fig Identification of *KI(klf6a-*HA*-P2A-gal4)* fish. (A) Expression pattern of *KI(klf6a-*HA*-P2A-gal4)*. White arrowheads indicate *klf6a* co-localization with vasculature. Blue arrowheads indicate *klf6a* expression in the lower branch in vascular loops. (B) Sequencing analysis of *KI(klf6a-*HA*-P2A-gal4)*. (C) Identification of *KI(klf6a-*HA*-P2A-gal4)* fish by PCR analysis.
(TIF)

S6 Fig *Klf6a* regulates zebrafish CV pruning. (A) Image of embryos injected with control MO or *klf6a* MO in the light field at 72 hpf. (B) Role of *klf6a* MO in zebrafish CV pruning. Boxes show enlarged images of the CV. The arrowhead indicates the unpruned vessel. Scale bar: 50 μm. (C) Quantification of vascular loops. Control MO: *n* = 11 embryos, *klf6a* MO: *n* = 27 embryos. *P* = 0.0204. Student’s unpaired two-tailed t test.
(TIF)

S7 Fig Generation of the *klf6a* mutant. (A) Diagrammatic representation of the deletion of the *klf6a* gene obtained by CRISPR/Cas9. The 7 bp DNA fragment deleted from exon 2 of the *klf6a* gene locus is shown in red, with the DNA sequence trace for the homozygous mutant shown underneath. (B) WISH of the *klf6a* gene of sibling and *klf6a*^*-/-*^ zebrafish at 36 hpf. Arrowheads indicate the CVP region. (C) Sequence for the *klf6a* mutant. (D) Image of the *klf6a* mutant identified by PCR analysis.
(TIF)

S8 Fig The effect of KLF6 on actin cytoskeleton *in vitro*. (A) KLF6 is located in the nuclei of HUVECs. Scale bar: 50 μm, 10 μm (enlarged images). (B) Immunofluorescence staining of siCTR-transfected and siKLF6-transfected HUVECs with VE-cadherin (green), phalloidin (red), and DAPI (blue). Dashed boxes show enlarged images of F-actin. Arrows indicate bundled F-actin closely flanking VE-cadherin-positive AJs. Arrowheads indicate increased stress fibers. Scale bar: 50 μm, 10 μm (enlarged images). (C) Immunofluorescence staining of siCTR-transfected and siKLF6-transfected HUVECs after treatment with 0 or 12 dyn/cm^2^ FSS for 24 h. Dashed boxes show enlarged images. Arrows indicate bundled F-actin closely flanking VE-cadherin-positive AJs. Arrowheads indicate increased stress fibers. Scale bar: 50 μm, 10 μm (enlarged images).
(TIF)

S9 Fig Junction remodeling and rearrangement of F-actin cytoskeleton in CV pruning. (A) and (B) Time-lapse live imaging of WT *Tg(Fliep*:*Lifeact-EGFP);KI(cdh5-mRFP)* embryos shows junction remodeling and rearrangement of actin cytoskeleton at multicellular to unicellular tube transformation stage (A) and at retraction stage (B) during CV pruning. (A) Cdh5-positve junction moves to the right (white arrowheads) and new junction gradually formed at narrow region (yellow arrowheads), in both of which F-actin forms and goes through similar rearrangement (white and yellow arrowheads). Meanwhile, F-actin depolymerized at non-narrow region (white arrow). Six vascular loops are taken time-lapse live imaging at the stage of stenosis. Scale bar: 25 μm. (B) F-actin retracts and gradually dissociates at retraction stage (white arrowheads). Four vascular loops are taken time-lapse live imaging at the stage of retraction. Scale bar: 10 μm.
(TIF)

S10 Fig Knockdown of *tagln2* impairs CV pruning. (A) Images of embryos after injection with pcDNA3.1-tagln2-EGFP or pcDNA3.1-tagln2-EGFP/*tagln2* MO. (B) Image of embryos injected with control MO or *tagln2* MO in the light field at 72 hpf. (C) Images of the CV in control or *tagln2* morphant. Boxes show enlarged images of the CV. Arrowheads indicate unpruned vessel. Scale bar: 50 μm. (D) Quantification of vascular loops. Control MO: *n* = 20 embryos, *tagln2* MO: *n* = 26 embryos. *P* = 0.0131. Student’s unpaired two-tailed t test.
(TIF)

S11 Fig Generation of the *tagln2* mutant. (A) Diagrammatic representation of the deletion of the *tagln2* gene obtained by CRISPR/Cas9. The 7 bp DNA fragment deleted from the exon 1 of the *tagln2* gene locus is shown in red, with the DNA sequence trace for the homozygous mutant shown underneath. (B) Sequence for the *klf6a* mutant. (C) Image of the *klf6a* mutant identified by PCR. (D) WISH of the *tagln2* gene of sibling and *tagln2*^*-/-*^ zebrafish at 36 hpf. Arrows indicate the CVP region.
(TIF)

S12 Fig Knockdown of TAGLN2 increases stress fibers *in vitro*. (A) Immunofluorescence staining of HUVECs with TAGLN2 (blue), anti-VE-cadherin (green), and phalloidin (red). Scale bar: 50 μm. (B) Immunofluorescence staining of SiCTR-transfected and siTAGLN2-transfected HUVECs. Dashed boxes show F-actin in areas that have been enlarged. Arrows indicate F-actin bundles closely flanking VE-cadherin-positive AJs. Arrowheads indicate increased stress fibers. Scale bar: 50 μm.
(TIF)

S1 Table MO sequences. (DOCX)

S2 Table Primers for the *klf6a* or *tagln2* mutant, *KI(klf6a—*HA*-P2A-gal4)* genotyping, WISH, ChIP-PCR, and RT-PCR. (DOCX)

S3 Table All antibodies used in the study. (DOCX)

S1 Video Quick scan of *Tg(flk1*:*EGFP;gata1a*:*dsRed)* embryos for calculating velocity of pruning branches at 48 hpf. (MP4)

S2 Video Quick scan of *Tg(flk1*:*EGFP;gata1a*:*dsRed)* embryos for calculating velocity of pruning branches at 51 hpf. (MP4)

S3 Video Quick scan of *Tg(flk1*:*EGFP;gata1a*:*dsRed)* embryos for calculating velocity of pruning branches at 53.5 hpf. (MP4)

S4 Video Quick scan of *Tg(flk1*:*EGFP;gata1a*:*dsRed)* embryos for calculating velocity of pruning branches at 56 hpf. (MP4)

S5 Video Quick scan of *Tg(flk1*:*EGFP;gata1a*:*dsRed)* embryos for calculating velocity of pruning branches at 58.5 hpf. (MP4)
