# Supplementary figures and images for: The blood flow-klf6a-tagln2 axis drives vessel pruning in zebrafish by regulating endothelial cell rearrangement and actin cytoskeleton dynamics
Source: PLoS Genet. 2021 Jul 28;17(7):e1009690. doi: 10.1371/journal.pgen.1009690 (PMC8318303; doi:10.1371/journal.pgen.1009690)

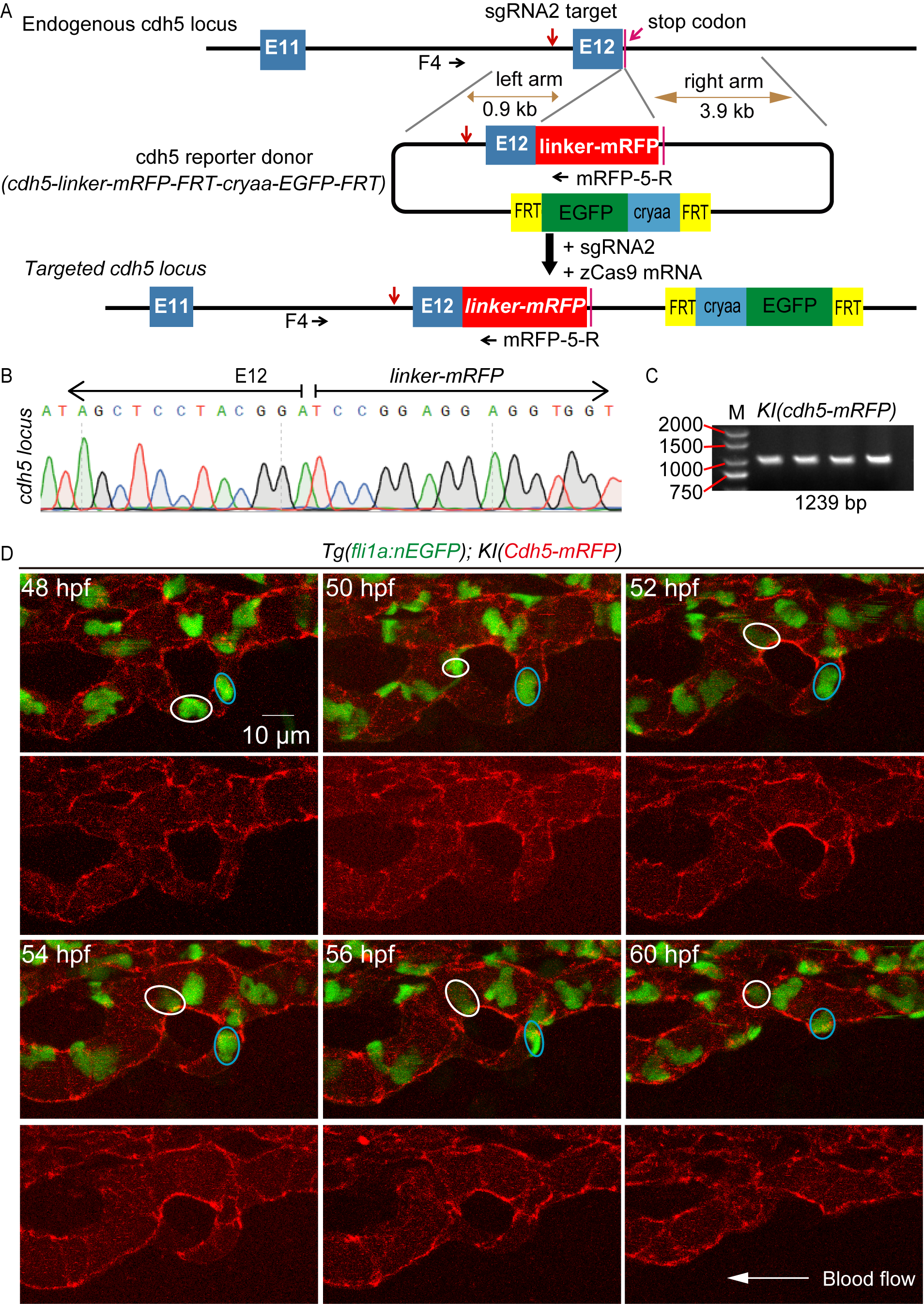

Supplement: S1 Fig — (A) Schematic diagram of KI(cdh5-mRFP) fish. (B) Sequence of KI(cdh5-mRFP) fish. (C) Identification of KI(cdh5-mRFP) by PCR analysis. (D) Time-lapse live imaging of Tg(fli1a:EGFP);KI(cdh5-mRFP) embryos shows EC rearrangement in CV pruning. The arrow shows the direction of the blood flow. Colored circles indicate the EC nuclei. Six time-lapse live imaging were taken. Scale bar: 10 μm. (TIF) [file pgen.1009690.s001.tif]

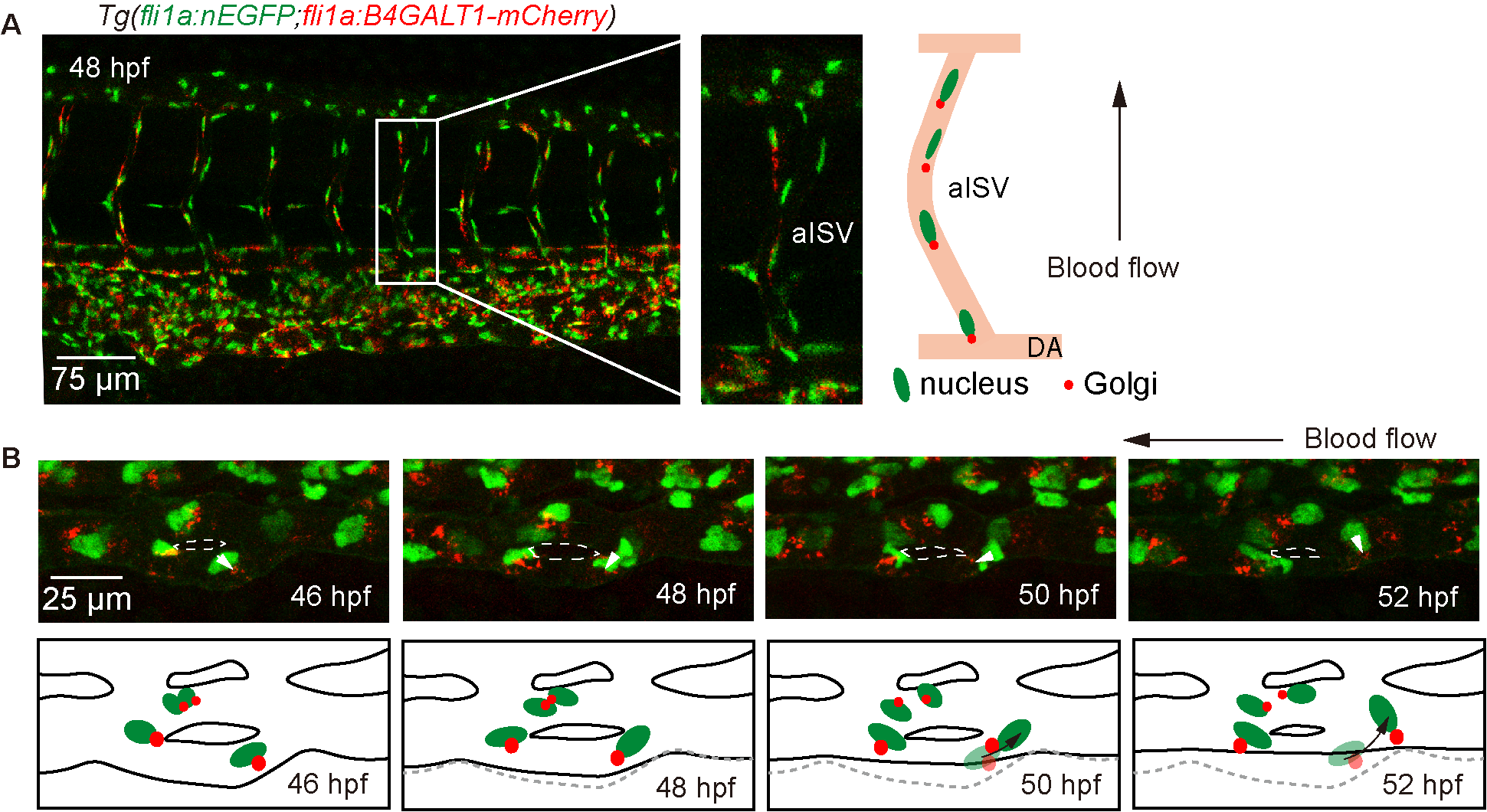

Supplement: S2 Fig — (A) Imaging of Tg(B4GALT1-mCherry; fli1a:nEGFP) embryos shows EC polarity against blood flow in intersegmental vessels (ISVs) at 48 hpf. The green indicates nucleus, the red indicates Golgi. Box indicates the enlarged image of Arterial ISV (aISV). The arrow indicates the direction of the blood flow. Scale bar: 75 μm. (B) EC polarity is not involved in CV pruning during EC migration. The arrow indicates the direction of the blood flow. Nine moves were analyzed. among 22 venous ECs, 19 ECs did not reveal polarities against the blood flow. Scale bar: 25 μm. (TIF) [file pgen.1009690.s002.tif]

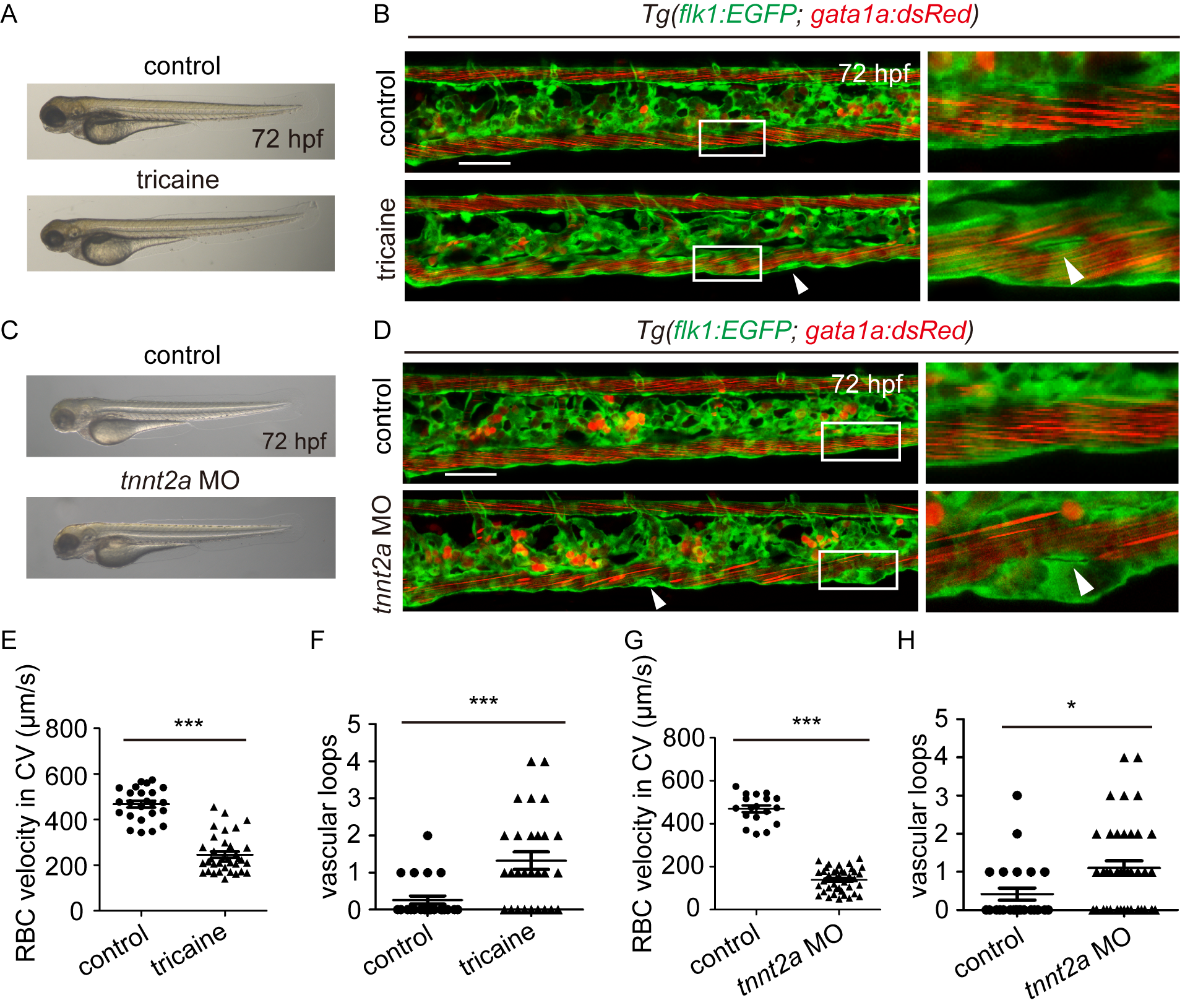

Supplement: S3 Fig — (A) and (C) The representative images of gross morphology in bright field at 72 hpf. (B) and (D) The representative image of CV pruning in zebrafish, and images were taken at 72 hpf. Boxes indicate the enlarged image of CV. Arrowheads indicate unpruned vessel. Scale bar: 50 μm. (A) and (B) Embryos treated with ddH2O or 0.06 mg/ml tricaine from 30 hpf to 72 hpf. (C) and (D) Embryos injected with 1ng control MO or 0.2 ng tnnt2a MO. (E) The RBC velocity in the control group and the group treated with tricaine. Six videos (4 RBCs/videos) and nine videos (4 RBCs/ video) were used to calculate RBC velocity in the control and tricaine treatment groups, respectively. P < 0.0001. (F) Quantification of vascular loops in the CV. Control: n = 23 embryos, tricaine treatment: n = 28 embryos. P = 0.0004. (G) The RBC velocity in the control group and the tnnt2a MO-injected group. 6 videos (3 RBCs/video) and 10 videos (4 RBCs/ video) were used to calculate RBC velocity in the control and tnnt2a morphant groups, respectively. P < 0.0001. (H) Quantification of vascular loops in the CV. Control: n = 24 embryos, tnnt2a morphant: n = 39 embryos. P = 0.0132. Student’s unpaired two-tailed t test. *P < 0.05, **P < 0.01, ***P < 0.001. (TIF) [file pgen.1009690.s003.tif]

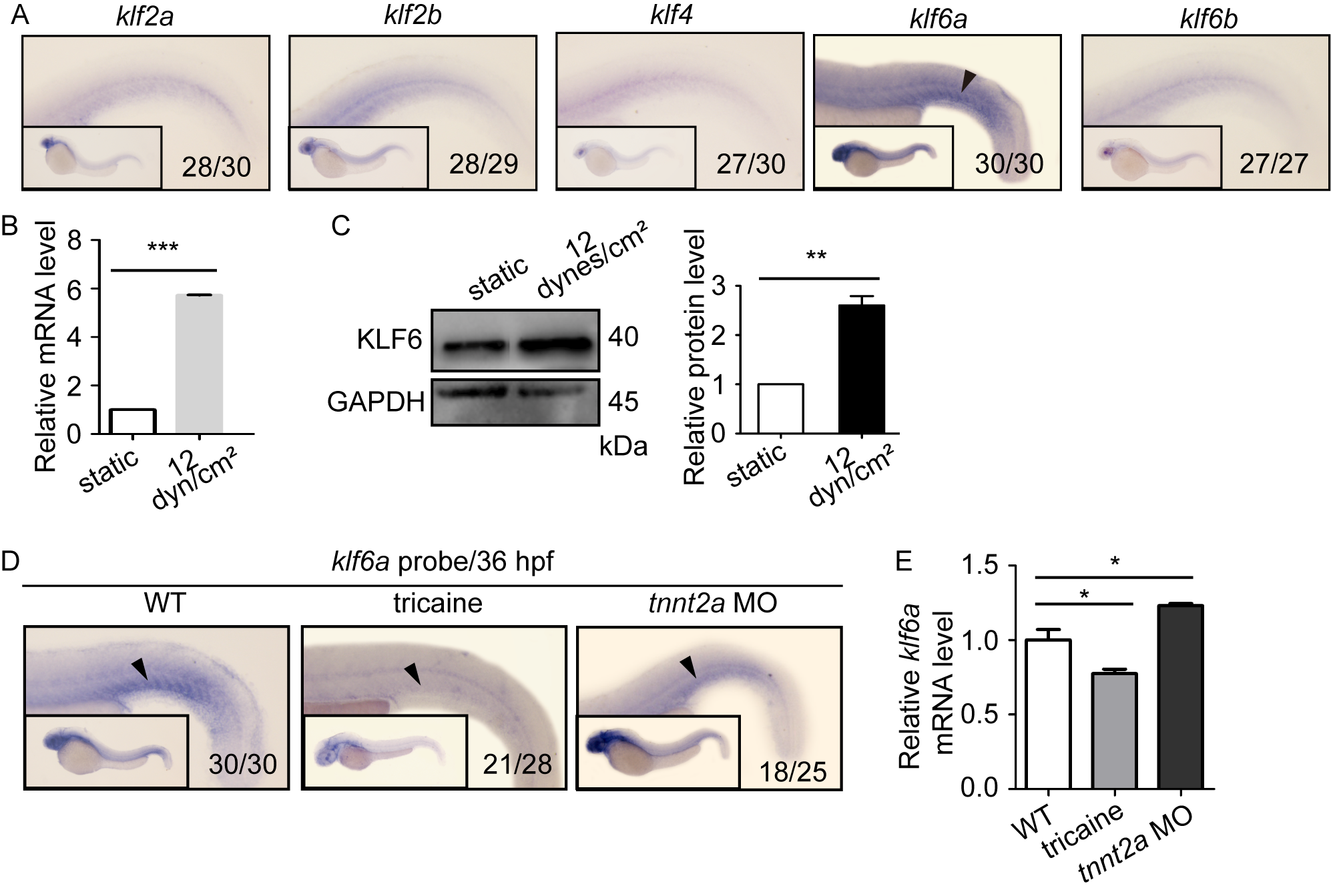

Supplement: S4 Fig — (A) WISH of the klf2a, klf2b, klf4, klf6a, and klf6b genes of zebrafish at 36 hpf. The arrowhead indicates a CVP with klf6a expression. (B) and (C) Relative mRNA (P < 0.0001) and protein (P = 0.0010) levels of KLF6 after treatment with 0 or 12 dyn/cm2 flow shear stress (FSS). Student’s unpaired two-tailed t test. **P < 0.01, ***P < 0.001. (D) WISH of the klf6a gene in zebrafish at 36 hpf after treatment with tricaine or tnnt2a MO. Arrowheads indicate the CVP region. (E) Quantification of klf6a relative mRNA level in zebrafish embryos at 36 hpf after treatment with tricaine (P = 0.0411) or tnnt2a MO (P = 0.0317). (TIF) [file pgen.1009690.s004.tif]

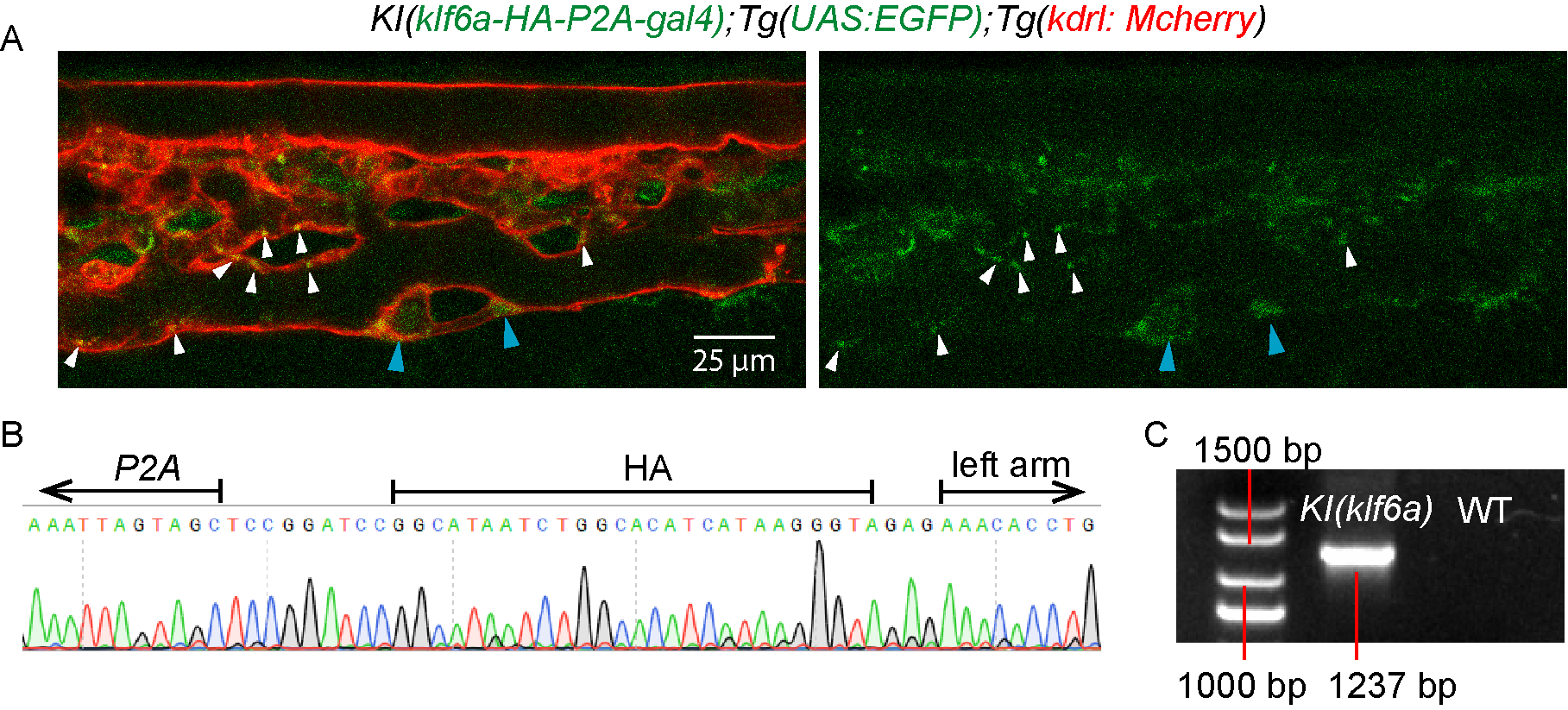

Supplement: S5 Fig — (A) Expression pattern of KI(klf6a-HA-P2A-gal4). White arrowheads indicate klf6a co-localization with vasculature. Blue arrowheads indicate klf6a expression in the lower branch in vascular loops. (B) Sequencing analysis of KI(klf6a-HA-P2A-gal4). (C) Identification of KI(klf6a-HA-P2A-gal4) fish by PCR analysis. (TIF) [file pgen.1009690.s005.tif]

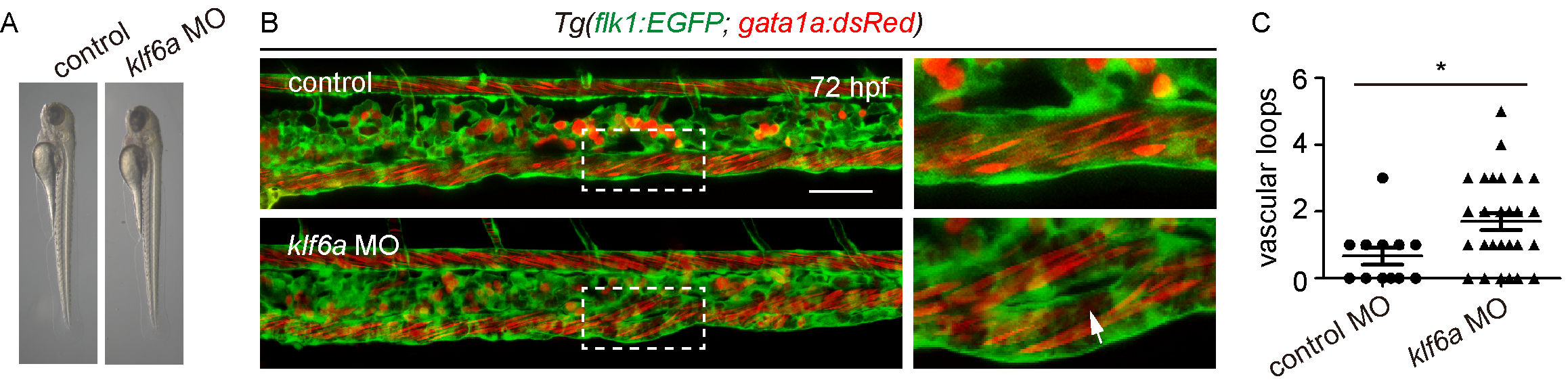

Supplement: S6 Fig — (A) Image of embryos injected with control MO or klf6a MO in the light field at 72 hpf. (B) Role of klf6a MO in zebrafish CV pruning. Boxes show enlarged images of the CV. The arrowhead indicates the unpruned vessel. Scale bar: 50 μm. (C) Quantification of vascular loops. Control MO: n = 11 embryos, klf6a MO: n = 27 embryos. P = 0.0204. Student’s unpaired two-tailed t test. (TIF) [file pgen.1009690.s006.tif]

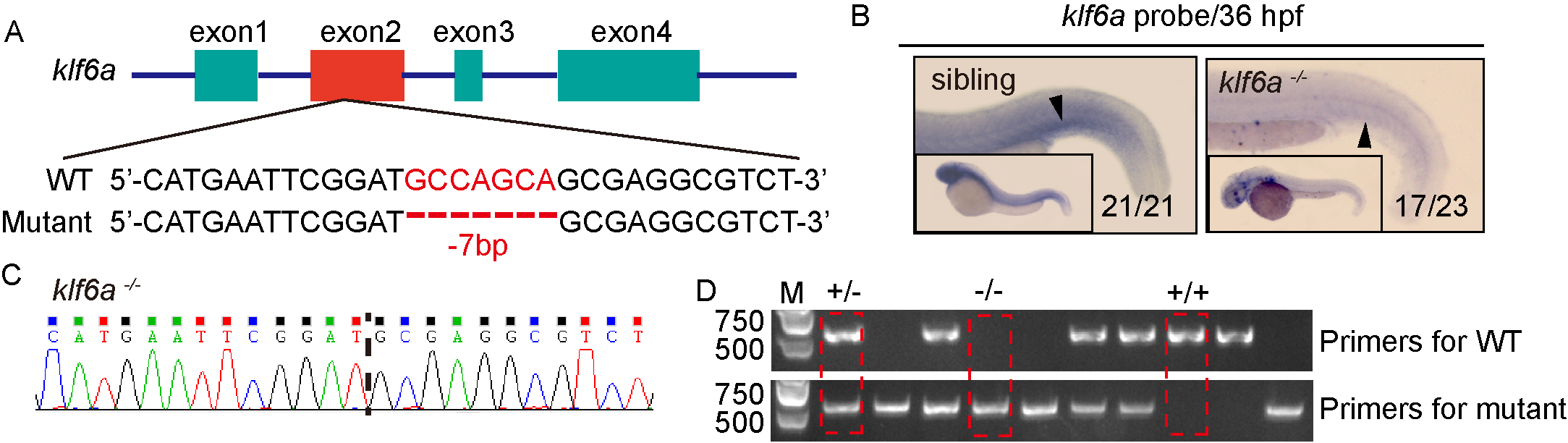

Supplement: S7 Fig — (A) Diagrammatic representation of the deletion of the klf6a gene obtained by CRISPR/Cas9. The 7 bp DNA fragment deleted from exon 2 of the klf6a gene locus is shown in red, with the DNA sequence trace for the homozygous mutant shown underneath. (B) WISH of the klf6a gene of sibling and klf6a-/- zebrafish at 36 hpf. Arrowheads indicate the CVP region. (C) Sequence for the klf6a mutant. (D) Image of the klf6a mutant identified by PCR analysis. (TIF) [file pgen.1009690.s007.tif]

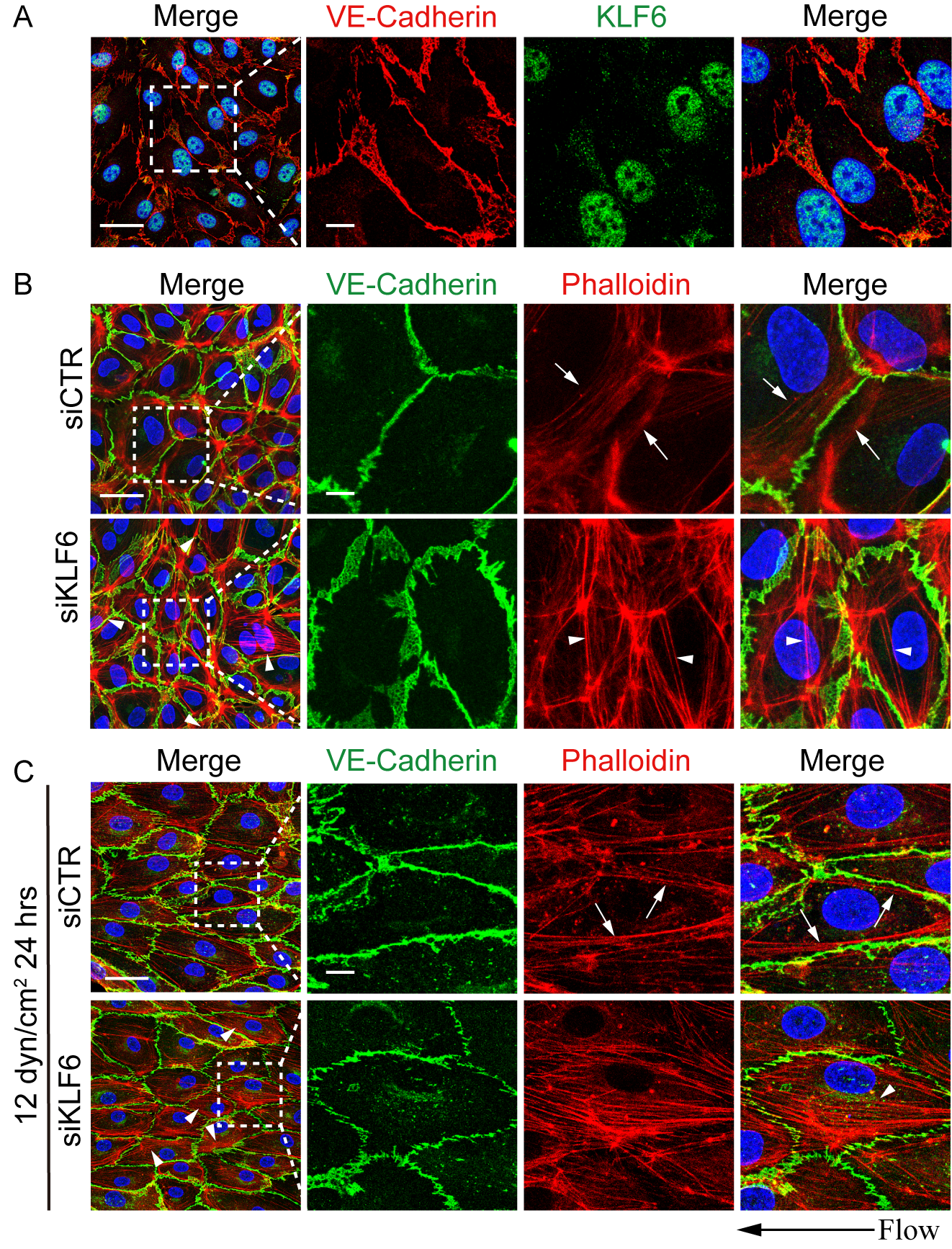

Supplement: S8 Fig — (A) KLF6 is located in the nuclei of HUVECs. Scale bar: 50 μm, 10 μm (enlarged images). (B) Immunofluorescence staining of siCTR-transfected and siKLF6-transfected HUVECs with VE-cadherin (green), phalloidin (red), and DAPI (blue). Dashed boxes show enlarged images of F-actin. Arrows indicate bundled F-actin closely flanking VE-cadherin-positive AJs. Arrowheads indicate increased stress fibers. Scale bar: 50 μm, 10 μm (enlarged images). (C) Immunofluorescence staining of siCTR-transfected and siKLF6-transfected HUVECs after treatment with 0 or 12 dyn/cm2 FSS for 24 h. Dashed boxes show enlarged images. Arrows indicate bundled F-actin closely flanking VE-cadherin-positive AJs. Arrowheads indicate increased stress fibers. Scale bar: 50 μm, 10 μm (enlarged images). (TIF) [file pgen.1009690.s008.tif]

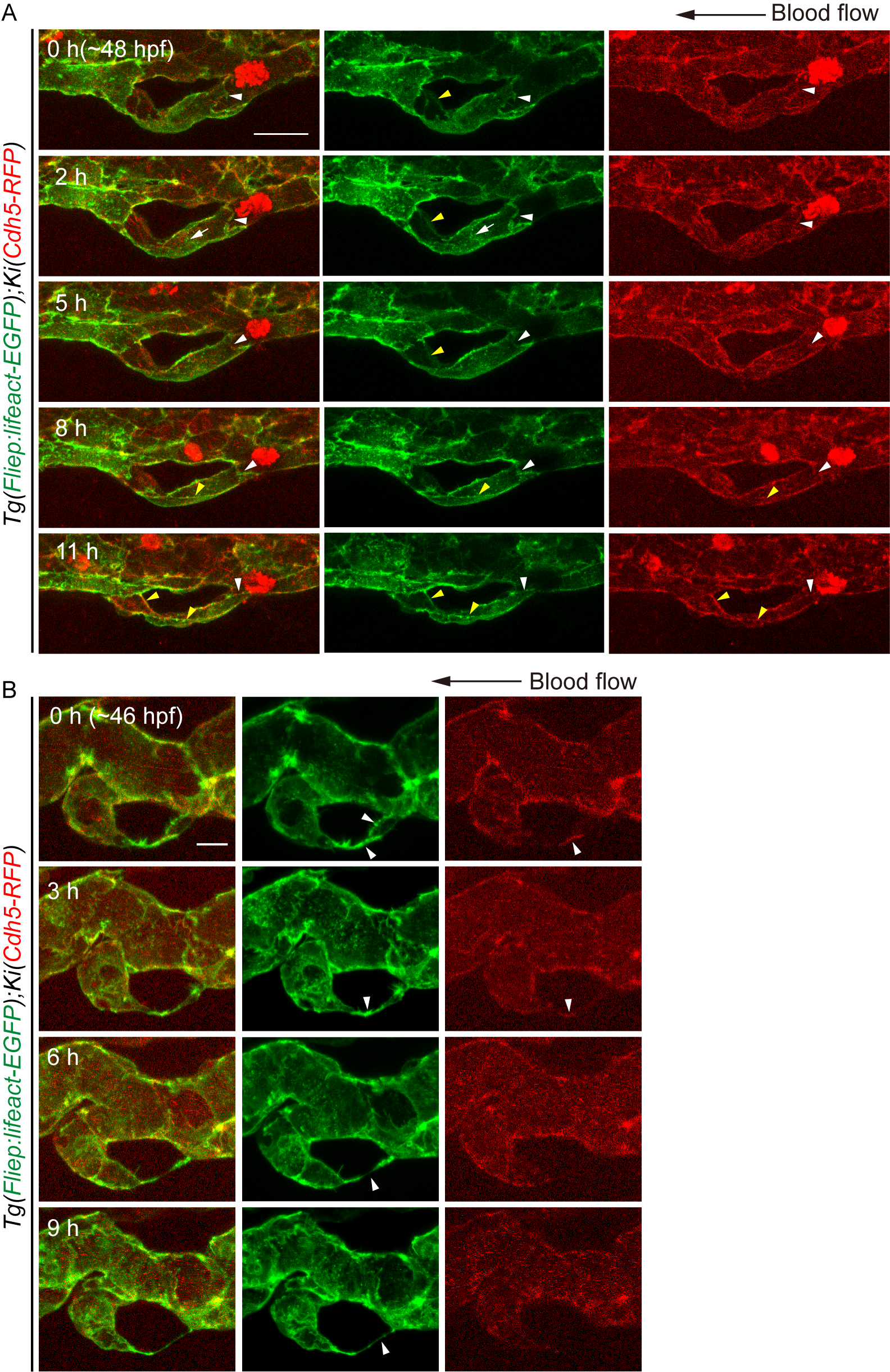

Supplement: S9 Fig — (A) and (B) Time-lapse live imaging of WT Tg(Fliep:Lifeact-EGFP);KI(cdh5-mRFP) embryos shows junction remodeling and rearrangement of actin cytoskeleton at multicellular to unicellular tube transformation stage (A) and at retraction stage (B) during CV pruning. (A) Cdh5-positve junction moves to the right (white arrowheads) and new junction gradually formed at narrow region (yellow arrowheads), in both of which F-actin forms and goes through similar rearrangement (white and yellow arrowheads). Meanwhile, F-actin depolymerized at non-narrow region (white arrow). Six vascular loops are taken time-lapse live imaging at the stage of stenosis. Scale bar: 25 μm. (B) F-actin retracts and gradually dissociates at retraction stage (white arrowheads). Four vascular loops are taken time-lapse live imaging at the stage of retraction. Scale bar: 10 μm. (TIF) [file pgen.1009690.s009.tif]

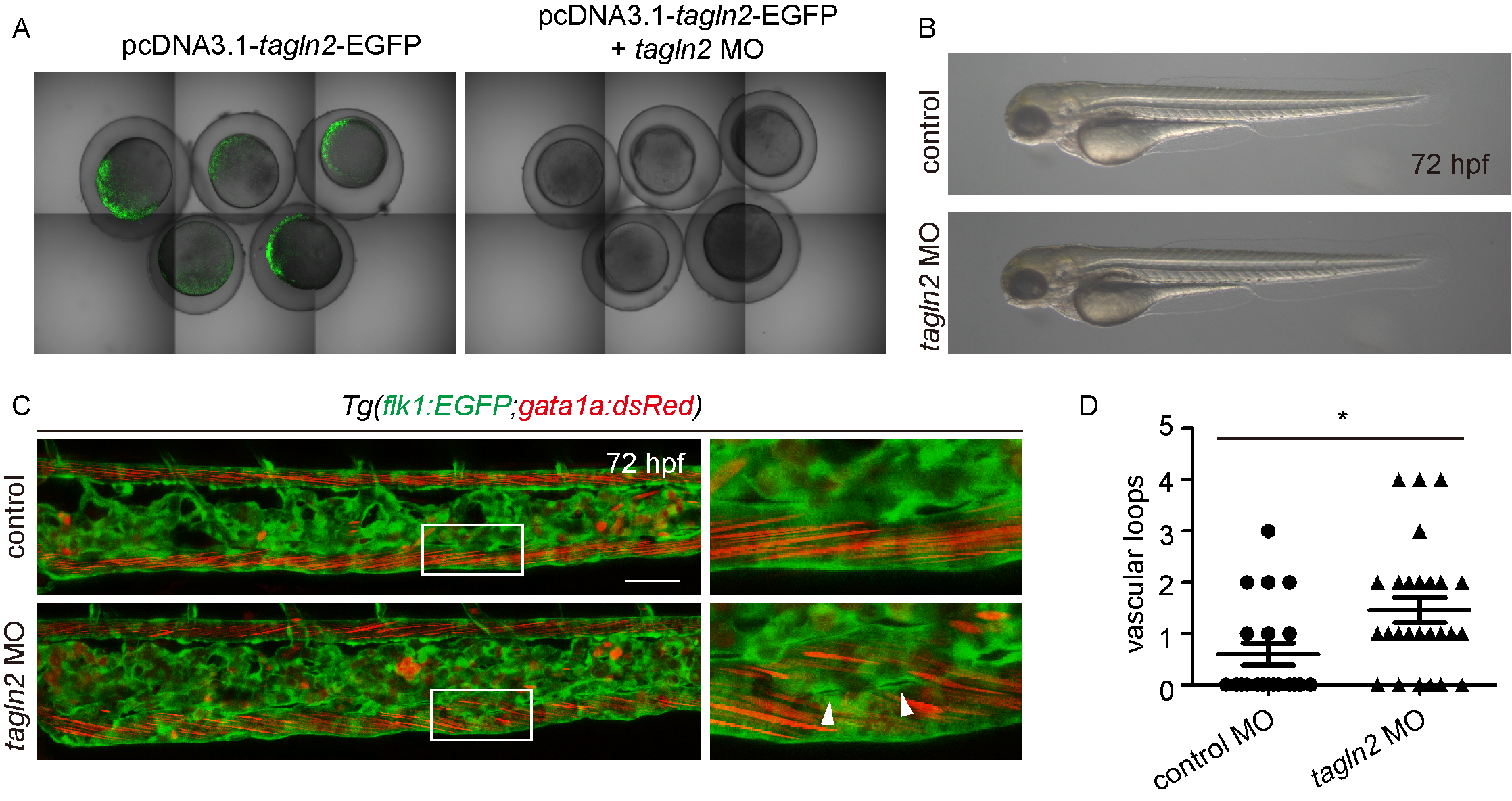

Supplement: S10 Fig — (A) Images of embryos after injection with pcDNA3.1-tagln2-EGFP or pcDNA3.1-tagln2-EGFP/tagln2 MO. (B) Image of embryos injected with control MO or tagln2 MO in the light field at 72 hpf. (C) Images of the CV in control or tagln2 morphant. Boxes show enlarged images of the CV. Arrowheads indicate unpruned vessel. Scale bar: 50 μm. (D) Quantification of vascular loops. Control MO: n = 20 embryos, tagln2 MO: n = 26 embryos. P = 0.0131. Student’s unpaired two-tailed t test. (TIF) [file pgen.1009690.s010.tif]

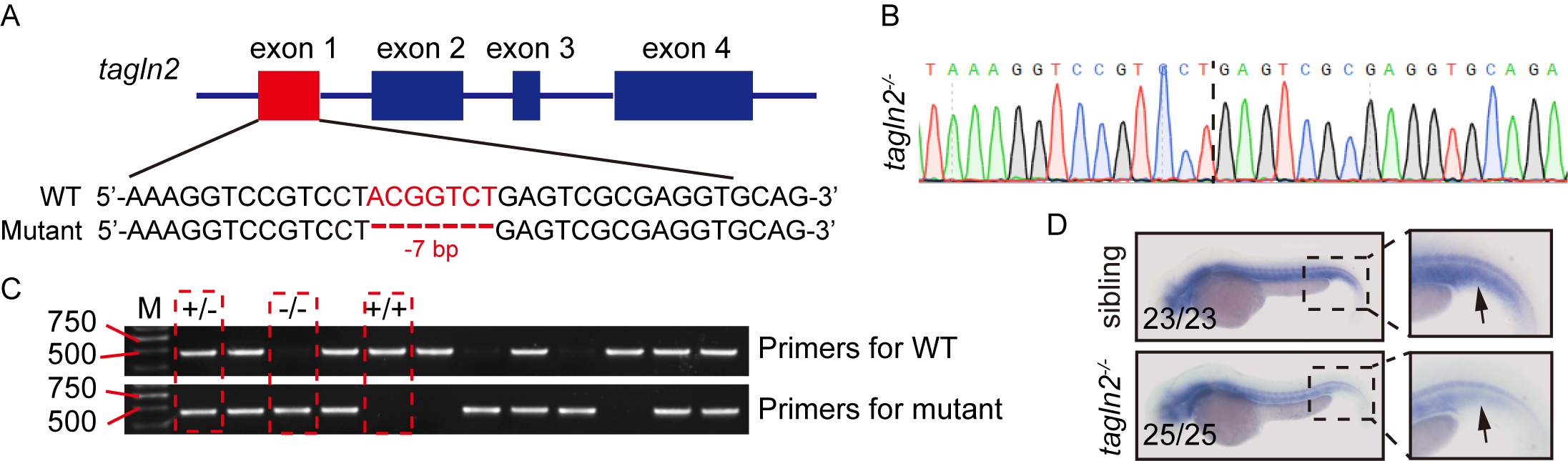

Supplement: S11 Fig — (A) Diagrammatic representation of the deletion of the tagln2 gene obtained by CRISPR/Cas9. The 7 bp DNA fragment deleted from the exon 1 of the tagln2 gene locus is shown in red, with the DNA sequence trace for the homozygous mutant shown underneath. (B) Sequence for the klf6a mutant. (C) Image of the klf6a mutant identified by PCR. (D) WISH of the tagln2 gene of sibling and tagln2-/- zebrafish at 36 hpf. Arrows indicate the CVP region. (TIF) [file pgen.1009690.s011.tif]

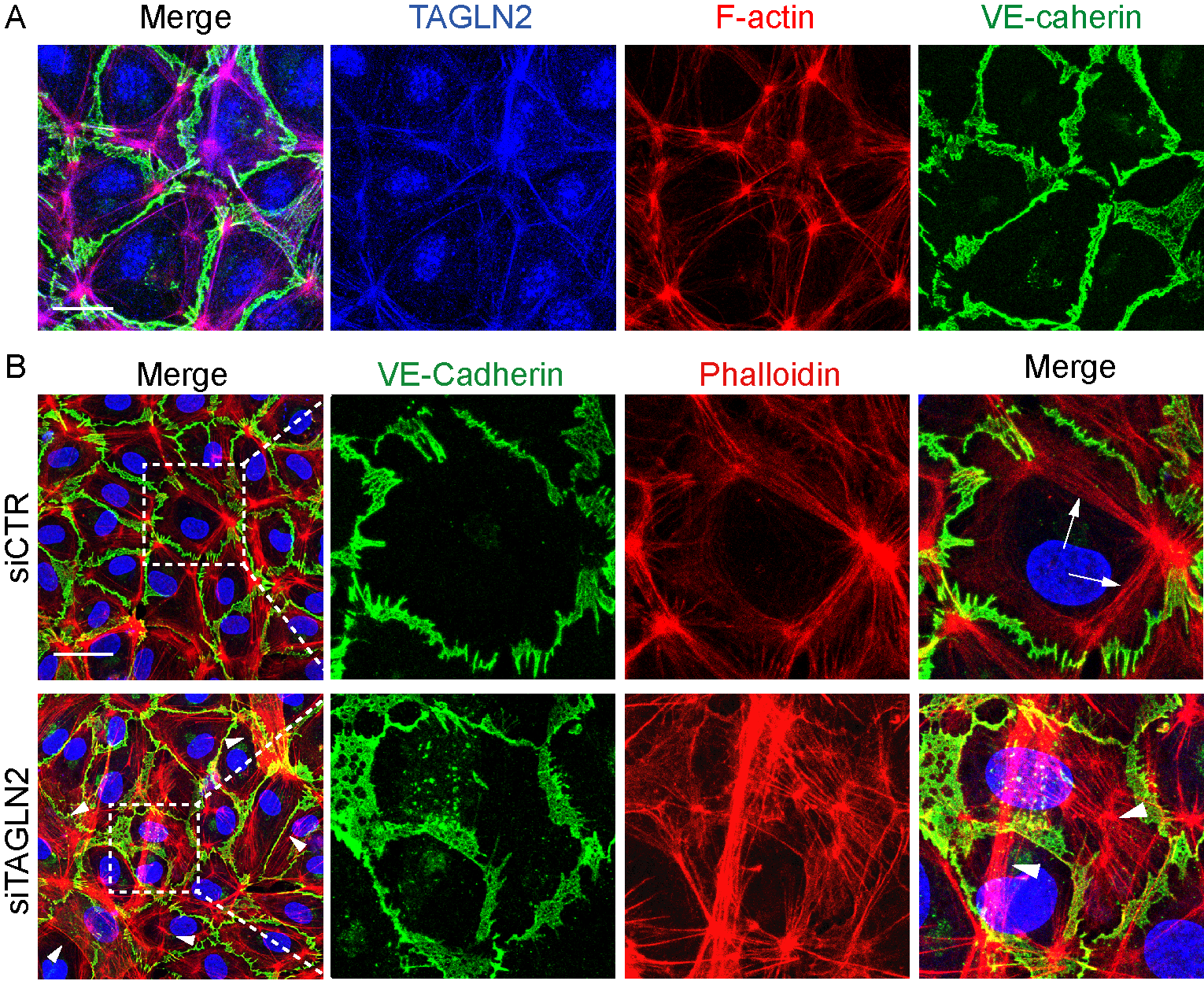

Supplement: S12 Fig — (A) Immunofluorescence staining of HUVECs with TAGLN2 (blue), anti-VE-cadherin (green), and phalloidin (red). Scale bar: 50 μm. (B) Immunofluorescence staining of SiCTR-transfected and siTAGLN2-transfected HUVECs. Dashed boxes show F-actin in areas that have been enlarged. Arrows indicate F-actin bundles closely flanking VE-cadherin-positive AJs. Arrowheads indicate increased stress fibers. Scale bar: 50 μm. (TIF) [file pgen.1009690.s012.tif]
