# Supplementary material for: The blood flow-klf6a-tagln2 axis drives vessel pruning in zebrafish by regulating endothelial cell rearrangement and actin cytoskeleton dynamics
Source: PLoS Genet. 2021 Jul 28;17(7):e1009690. doi: 10.1371/journal.pgen.1009690 (PMC8318303; doi:10.1371/journal.pgen.1009690)
Supplement: S1 Table — (DOCX) [file pgen.1009690.s013.docx]

**S1 Table. MO sequences.**

| Gene | MO sequence |
| --- | --- |
| *tnnt2a*^42^ | 5′-CATGTTTGCTCTGATCTGACACGCA-3′ |
| *klf6a*^22^ | 5′-CACATTGGTAGAACATCCATTGCAA-3′ |
| *tagln2* | 5′-GGACGGACCTTTATTTGCCATTTTG-3′ |
| Control MO^4^ | 5′-CCTCTTACCTCAGTTACAATTTATA-3′ |
