# Supplementary material for: The blood flow-klf6a-tagln2 axis drives vessel pruning in zebrafish by regulating endothelial cell rearrangement and actin cytoskeleton dynamics
Source: PLoS Genet. 2021 Jul 28;17(7):e1009690. doi: 10.1371/journal.pgen.1009690 (PMC8318303; doi:10.1371/journal.pgen.1009690)
Supplement: S2 Table — (DOCX) [file pgen.1009690.s014.docx]

**S2 Table. Primers for the *klf6a* or *tagln2* mutant, *KI(klf6a-*HA*-P2A-gal4)* genotyping, WISH, ChIP-PCR, and RT-PCR.**

| Purpose | Primer name | Primer sequence |
| --- | --- | --- |
| *klf6a* mutant genotyping | WT-F | 5′-GGATGCCAGCAGCGAGG-3′ |
|  | Mutant-F | 5′-TGAATTCGGATGCGAGG-3′ |
|  | Common-R | 5′-TGCAGGAGACGTGAAACCT-3′ |
| *tagln2* mutant genotyping | WT-F | 5′-CGTCCTACGGT CTGAGTCG-3′ |
|  | Mutant-F | 5′-AAAGGTCCGTCCTGAGTC -3′ |
|  | Common-R | 5′- CTCTCACCCTCCCATAAGTCG -3′ |
| WISH | *klf2a*-F | 5′-GGATCCGGAAGGATGAACTGGACAGG-3′ |
|  | *klf2a*-R | 5′-TAATACGACTCACTATAGGGCTCGAGGCGTTTAGTCCACATTTTCCA-3′ |
|  | *klf2b*-F | 5′-GAATTCCGCACACAATTGGTCTAGGA-3′ |
|  | *klf2b*-R | 5′-TAATACGACTCACTATAGGGTACGTACATCGTTGTGCATTTTCCAC-3′ |
|  | *klf4*-F | 5′-CCCAGATATCAGCGACGTTT-3′ |
|  | *klf4*-R | 5′-TAATACGACTCACTATAGGGCCTGCGGAAATCCAGAATAA-3′ |
|  | *klf6a*-F | 5′-TCCCACTCACATCTTTACCTCC-3′ |
|  | *klf6a*-R | 5′-GAATTGTAATACGACTCACTATAGGTTTCCCGACACCATTCAGCC-3′ |
|  | *klf6b*-F | 5′-TGTGCTTGACACGGGATAC-3′ |
|  | *klf6b*-R | 5′-TAATACGACTCACTATAGGGACCTCTATTCAGCACCAGAAC-3′ |
|  | *tagln2*-F | 5′-CTCACTGCTGCCGAACGATA-3′ |
|  | *tagln2*-R | 5′-TAATACGACTCACTATAGGGACTGGCTCATGTGAGGGGTA-3′ |
| qPCR | KLF6-F | 5′-TCTCATCAGCCCGAGCTTTTG-3′ |
|  | KLF6-R | 5′-GAGCTGTCAGAGGATTCGCT-3′ |
|  | GAPDH-F | 5′-TGCACCACCAACTGCTTAGC-3′ |
|  | GAPDH-R | 5′-GGCATGGACTGTGGTCATGAG-3′ |
|  | β-actin-F | 5′-CTGTCTTCCCATCCATCGTGGGTC-3′ |
|  | β-actin-R | 5′-CTCCATATCATCCCAGTTGGTGACA-3′ |
|  | TAGLN2-F | 5′-TCCAGAACTGGCTCAAGGATG-3′ |
|  | TAGLN2-R | 5′-TCCCAGAGGTCCACAGTTTG-3′ |
| ChIP-PCR | Tagln2 ChIP-F1 | 5′-GTCCATGACAACAGGGACTG-3′ |
|  | Tagln2 ChIP-R1 | 5′-TCTTGGAGTAGTCAGTGAATAAACT-3′ |
|  | Tagln2 ChIP F2 | 5′-TATGCGCTATGTCGCGGTAA-3′ |
|  | Tagln2 ChIP R2 | 5′-AAGGAAATGTCACGCCAGCA-3′ |
| *KI(klf6a*-HA-*P2A-gal4*) identification | *KI(klf6a)* F2 | 5′-CCTGCTTGTATTTCACGTACAGA-3′ |
|  | *KI(klf6a)* gal4 R2 | 5′-GGAGAGTAGCGACACTCCCAGT-3′ |
| *KI(Cdh5-mRFP)* identification | *cdh5-F4* | GGATAAACCATTTCAGTTGTTGCTCC |
|  | *mRFP-5-R* | CTCGGAGGAGGCCATTGAACC |
