# Supplementary material for: The blood flow-klf6a-tagln2 axis drives vessel pruning in zebrafish by regulating endothelial cell rearrangement and actin cytoskeleton dynamics
Source: PLoS Genet. 2021 Jul 28;17(7):e1009690. doi: 10.1371/journal.pgen.1009690 (PMC8318303; doi:10.1371/journal.pgen.1009690)
Supplement: S3 Table — (DOCX) [file pgen.1009690.s015.docx]

**S3 Table. All antibodies used in the study.**

| Antibody | Company | Catalog No. |
| --- | --- | --- |
| Phalloidin | Cell Signaling Technology | 12877 |
| VE-cadherin | Santa Cruz | sc-9989 |
| KLF6 | Thermo Fisher Scientific | PA5-79560 |
| TAGLN2 | ABclonal | A6761 |
| Donkey anti-mouse IgG H&L (Alexa Fluor 488) | Abcam | ab150105 |
| Myc-Tag (9B11) mouse mAb | Cell Signaling Technology | 2276 |
| β-actin | Cell Signaling Technology | 3700 |
| PAGDH | Cell Signaling Technology | 5174 |
